# GDF-15: A Potential Biomarker and Therapeutic Target in Systemic Lupus Erythematosus

**DOI:** 10.3389/fimmu.2022.926373

**Published:** 2022-07-14

**Authors:** Wang-Dong Xu, Qi Huang, Chan Yang, Rong Li, An-Fang Huang

**Affiliations:** ^1^ Department of Evidence-Based Medicine, Southwest Medical University, Luzhou, China; ^2^ Department of Rheumatology and Immunology, Affiliated Hospital of Southwest Medical University, Luzhou, China

**Keywords:** growth differentiation factor 15, lupus, polymorphism, inflammation, autoimmunity

## Abstract

Systemic lupus erythematosus (SLE) is a rheumatic disease. Growth differentiation factor 15 (GDF-15) is a member of transforming growth factor-β superfamily. To date, association of GDF-15 with SLE pathogenesis is not clarified. This study discussed GDF-15 serum levels and gene polymorphisms in SLE patients and lupus mouse model further demonstrated the role of GDF-15 in lupus development. We conducted two independent case-control studies for SLE patients. One is to evaluate serum levels of GDF-15 in 54 SLE patients and 90 healthy controls, and the other one is to analyze gene polymorphisms of GDF-15 in 289 SLE patients and 525 healthy controls. Serum levels of GDF-15 were detected by ELISA. GDF-15 gene polymorphisms (rs1055150, rs1058587, rs1059519, rs1059369, rs1227731, rs4808793, and rs16982345) were genotyped by the Kompetitive Allele-Specific PCR (KASP) method. Addition of recombinant GDF-15 into pristane-induced lupus mice evaluated histological and serological changes. Results showed that serum levels of GDF-15 were overexpressed in SLE patients and associated with disease activity. Polymorphisms rs1055150, rs1059369, rs1059519, and rs4808793 of GDF-15 gene were related to SLE risk. Lupus mice showed splenomegaly, severe histological scores, and high levels of autoantibodies [antinuclear antibodies (ANA) and total immunoglobulin G (IgG)], whereas administration of GDF-15 into lupus mice reduced the histological changes. Percentages of CD8^+^, CD11b^+^, CD19^+^, CD11C^+^ cells, T_H_2 cells, and pro-inflammatory cytokines (IL-1β, IL-2, IL-4, IL-21, and IL-22) were reduced after GDF-15 treatment in lupus mice. In conclusion, GDF-15 was related to lupus pathogenesis and inhibited lupus development.

## Introduction

Systemic lupus erythematosus (SLE) is a rheumatic autoimmune disease with heterogeneous clinical manifestations. To date, etiology and pathophysiology of SLE are not fully clarified (1). Glucocorticoids and immunosuppressive drugs are non-specific therapeutic agents for SLE patients, which may lead to adverse effects (2). Therefore, searching for potential biomarkers for SLE to better prevent and early diagnose this disease and targeting the biomarkers for therapy of SLE are urgently needed.

Previous studies found many target genes related to SLE risk, such as signal transducer and activator of transcription 4 (STAT4), death-associated protein 1 (DAP1), mannose binding lectin 2 (MAB2), prostaglandins E2 (PGE2), and PIN2/TERF1-interacting telomerase inhibitor 1 (PINX1) (3–8). Growth differentiation factor 15 (GDF-15) is a member of transforming growth factor–β (TGF-β) superfamily. GDF-15 is a 25-kDa homodimer, consisting of two 112–amino acid polypeptide chains (9). In physiological conditions, expression of serum GDF-15 is modest (10), whereas it is elevated in pathological conditions, such as in patients with renal failure, chronic liver disease, and rheumatoid arthritis (RA), suggesting that GDF-15 may be a potential biomarker for diseases (9, 11). To date, the role of GDF-15 in SLE is largely unknown, although limited studies with small sample size indicated high expression of GDF-15 in SLE patients (12, 13). However, whether GDF-15 gene polymorphisms relate to SLE risk in Chinese population and whether GDF-15 regulates lupus development need to be discussed. In this study, we first discussed the serum levels of GDF-15 in SLE patients and the association of GDF-15 gene polymorphisms with SLE risk in a Chinese Han population. Second, we conducted a lupus mouse model to confirm the functional role of GDF-15 in lupus development. Last, this study revealed the potential of serum GDF-15 as a SLE biomarker and demonstrated the role of GDF-15 in inhibiting lupus pathogenesis.

## Materials and methods

### Patients and Controls

There are two independent case-control studies. The first one is to evaluate the serum levels of GDF-15 in SLE patients and discuss the association of GDF-15 and SLE pathogenesis, including 54 SLE patients and 90 healthy controls (sex and age matched). The second one is to discuss the association of GDF-15 gene polymorphisms in 289 SLE patients and 525 healthy controls. Clinical and laboratory features of patients with SLE were collected, mainly including arthritis, discoid rash, alopecia, oral ulcers, vasculitis, C3, C4, erythrocyte sedimentation rate (ESR), C-reactive protein (CRP), immunoglobulin G (IgG), IgA, IgM, and rheumatoid factor (RF). Activity of SLE was calculated by SLE Disease Activity Index version 2000 (SLEDAI-2K), which was divided into less active disease activity (SLEDAI < 10) and high (active) disease activity (SLEDAI ≥ 10) (14). Diagnosis of SLE patients was according to the 1997 American College of Rheumatology (ACR) criteria for SLE (15). Patients were from Department of Rheumatology and Immunology, Affiliated Hospital of Southwest Medical University. Healthy controls were from Physical Examination Center of Jiangyang District Center for Disease Control and Prevention in Luzhou, Sichuan, China. This study was admitted by Ethic Research Committee of Affiliated Hospital of Southwest Medical University, and each participant agreed and signed informed consent. The study was carried out according to The Code of Ethics of the World Medical Association (Declaration of Helsinki).

### Detecting Serum Levels of GDF-15 in SLE Patients and ANA, Anti–Double-stranded DNA, Total IgG in Mice Models

Peripheral vein blood of each participant was collected and centrifuged, and serum was refrigerated at −80°C for further usage. Serum GDF-15 levels were detected by enzyme-linked immunosorbent assay (ELISA) kit (Cusabio, Houston, USA), according to the instruction of the manufacturer. Briefly, serum samples and different concentrations of standards were added to each well, covered with the adhesive strip. After incubating for 2 h at 37°C, the liquid was removed, added biotinylated antibody, and then incubated at 37°C for 1 h. After washing three times, the horseradish peroxidase–avidin was added into each well (incubating at 37°C for 1 h). After washing five times, 90 μl of TMB substrate was added and incubated at 37°C for 30 min without light. Finally, 50 ml of stop solution was added and determined optical density in microplate reader at 450 nm. The minimum detectable concentration is 1.95 pg/ml. Detecting levels of antinuclear antibodies (ANA), anti–double-stranded DNA (anti-dsDNA), and total IgG in serum from mice models was described as previously by ELISA kits (CUSABIO, Wuhan, China) (16). The minimum detecting level of ANA, anti-dsDNA, and total IgG was 7.8 pg/ml, 1.56 ng/ml, and 125 ng/ml, respectively. All samples were measured in duplicate.

### Single-Nucleotide Polymorphism Selecting and Genotyping

Previous studies were systematically reviewed, and the National Center for Biotechnology Information database (NCBI; https://www.ncbi.nlm.nih.gov/) and Genome Reference Consortium Human Build 38 project (GRCh38; http://may2017.archive.ensembl.org/Homo_sapiens/Info/Index) were considered. Finally, seven GDF-15 gene polymorphisms (rs1055150, rs1058587, rs1059519, rs1059369, rs1227731, rs4808793, and rs16982345) were selected. All the loci ware according with pairwise tagging of HapMap population with r^2^ ≥ 0.8, a minor allele frequency ≥5%, and Chinese Han Beijing (CHB) ethnicity. Genomic DNAs were extracted and purified from peripheral blood mononuclear cells by TIANamp Blood DNA kits (Tiangen, Beijing, China). Genotyping was conducted by the Kompetitive Allele-Specific PCR (KASP) method (Gene Company, Shanghai, China). Primer information for each polymorphism was listed in **Supplementary Table 1**.

### Mice Models and Experimental Protocol

Female C57BL/6 mice (6–8 weeks) were purchased from SiPeiFu Biotechnology (Beijing, China). Mice were kept with water and food freely (temperature, 23 ± 1°C; humidity, 50 ± 10%, 12/12-h light/dark cycle). Mice were randomly divided into six groups (seven mice per group). Five groups of mice were intraperitoneally injected with 0.5 ml of pristane (Sigma Aldrich, St Louis, USA) to induce lupus. Another group was injected with phosphate-buffered saline solution (PBS). Recombinant human GDF-15 protein (SinoBiological, Beijing, China) was dissolved into PBS for further usage. At week 19, five groups of mice injected with pristane were further injected with PBS, or 10, or 50, or 100, or 500 μg/kg of GDF-15 every other day (seven times), respectively. The mice treated with PBS were further injected with PBS every other day (seven times). At week 23, all the mice were euthanized. Heart blood was collected and centrifuged for obtaining serum. The liver, spleen, and two kidneys were weighed and photographed. Studies related to mice were approved from Animal Ethics Committee of Southwest Medical University.

### Histology and Scoring

Kidneys were fixed in 10% formalin for 24 h. Then, kidneys were embedded in paraffin and cut into slices (4 μm). Finally, sections were stained with hematoxylin and eosin (HE) to evaluate the severity of glomerular lesions. Ponceau, Fuchsin, and Aniline blue (Masson) staining assessed renal fibrosis. Immunofluorescence assay evaluated immune complex (total IgG) deposition. HE and Masson scoring was conducted according to previous studies (17–19). HE staining assessed renal injury from five dimensions, including glomerular injury, tubular injury, renal interstitial inflammation, renal interstitial fibrosis, and protein tubule. Based on staining area, all five dimensions can be divided into none (<5%), mild (5%–25%), moderate (26%–50%), and severe (>50%), scored 0–3, respectively. The sum of five renal lesion scores was the HE staining score (**Supplementary Table 2**). For Masson trichrome staining, every kidney slice was stained with Aniline blue, Acid Fuchsin, and Ponceau S, where the collagen fibers, mucus, and cartilage are blue. Muscle fibers, cellulose, muscle, glia, and cytoplasm are red; red blood cells are orange; and nuclei are black blue. Masson score is the percentage of the staining area in the total visual field area under the 400× microscope. The average of three random fields in every mouse kidney slice is the final Masson score.

### Inflammatory Cytokines Microarray

Eighteen inflammatory cytokines [IL-1 beta (IL-1β), IL-2, IL-4, IL-5, IL-6, IL-10, IL-12p70, IL-13, IL-17A, IL-17F, IL-21, IL-22, IL-23, IL-28A (IFN-lambda 2), interferon-gamma (IFNγ), macrophage inflammatory protein-3 alpha (MIP-3α), TGF-β1, and tumor necrosis factor–alpha (TNF-α)] were tested by microarray (RayBiotech, Georgia, USA) according to the manufacturer’s instruction. Briefly, slides were blocked with 100 µl of sample diluent at room temperature (30 min), and then added 100 µl of standard cytokines or samples in each well, incubating at room temperature for 2 h. Liquid was removed, washed seven times, and incubated with biotinylated antibody cocktail for 1.5 h. Then, slides were washed five times, adding Cy3 equivalent dye-conjugated streptavidin to each well for 1 h without light. Finally, fluorescence was detected by Axon GenePix 4400B Microarray Scanner (Molecular Devices, Sunnyvale, USA). Data were analyzed by GenePix Pro 6.0 software (Axon Instruments, Foster City, USA).

### Cell Isolation and Flow Cytometry

Mouse spleen was collected, and ground and red blood cells were lysed (Beyotime, Shanghai, China). After obtaining leukocytes, cells were used to detect proportion of CD14^+^, CD11c^+^, CD11b^+^, CD19^+^, CD3^+^, CD8^+^, CD4^+^IFNγ^+^ (T_H_1), CD4^+^IL-4^+^ (T_H_2), CD4^+^IL-17A^+^ (T_H_17), and CD4^+^Foxp3^+^ (T_reg_) cells. The following antibodies were used for cytometry: fluorescein isothiocyanate–conjugated anti-CD3 (145-2C11), CD4 (GK1.5), CD11c (HL3), CD14 (rmC5-3); phycoerythrin (PE)–conjugated anti-Foxp3 (MF23); PE-CF594–conjugated anti-IFNγ (XMG1.2); allophycocyanin (APC)-conjugated anti-CD8 (53-6.7), CD19 (1D3), IL-4 (11B11), CD11b (M1/70); and APC-CyTM7–conjugated anti–IL-17A (TC11-18H10). All the antibodies were purchased from BD Biosciences (California, USA), and fixation buffer and permeabilization buffer were purchase from R&D Systems (Minneapolis, USA). Results were acquired by FACSVerse (BD Biosciences, Franklin Lakes, USA) and data were analyzed by FlowJo software (Tree Star Inc., Franklin Lakes, USA).

### Statistics

SPSS 26.0 and GraphPad Prism 8.0 were used for statistics. Data were described by means ± standard deviation or median (interquartile range). Receiver operating characteristic (ROC) analysis explored the diagnostic ability of serum GDF-15. Sensitivity, specificity, positive and negative likelihood ratio (+LR and −LR), Youden’s index, accuracy, and positive and negative predictive value (PPV and NPV) were calculated to evaluate capacity of serum GDF-15 in discriminating SLE from healthy controls. Spearman’s rank correlation test discussed the relationship between different parameters. Bonferroni test was used to control the probability of committing a type I error when we performed multiple comparisons. Odds ratio (OR) and 95% confidence interval (CI) were calculated by a logistic regression model. Power was analyzed by G*Power 3.1.9.2 (Franz Faul, Kiel University, Germany).

## Results

### Association of Serum GDF-15 in SLE Patients, and Potential to be A Narker for SLE

**Figure 1:**
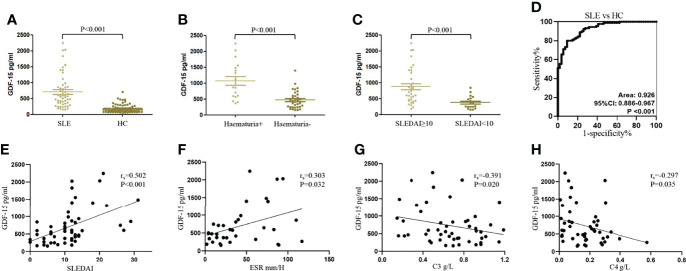
Increased serum levels of GDF-15 in SLE patients in training cohort. **(A)** Serum levels of GDF-15 in 54 systemic lupus erythematosus (SLE) patients and 90 healthy controls were detected by enzyme-linked immunosorbent assay. **(B, C)** GDF-15 expression in SLE patients with hematuria, different SLE disease activity index (SLEDAI). **(D)** Receiver operating characteristic (ROC) curve analysis of serum GDF-15 for diagnosis of SLE. **(E–H)** Association of serum GDF-15 with SLEDAI, erythrocyte sedimentation rate (ESR), C3, and C4. Mann–Whitney U-test discussed differences between two groups. Spearman’s non-parametric test calculated the association of parameters.

Characteristics of patients with SLE and healthy controls were shown in **Table 1**. Serum levels of GDF-15 in 54 SLE patients were higher as compared to that in 90 healthy controls [565.397 (334.924–887.646) vs. 153.641 (99.583–196.645) pg/ml, P < 0.001; **Figure 1A**]. SLE patients with hematuria had higher serum levels of GDF-15 than that in patients without hematuria [904.140 (589.491–1498.938) vs. 452.440 (253.513–691.203), P < 0.001; **Figure 1B**], and patients with high disease activity (SLEDAI ≥ 10) had much higher serum levels of GDF-15 than that in patients with less active disease activity (SLEDAI < 10) [727.972 (445.530–1374.793) vs. 306.506 (230.665–515.959) pg/ml, P < 0.001; **Figure 1C**]. Serum GDF-15 distinguished SLE patients from healthy controls with area under the curve 0.926 (95% CI: 0.886–0.967, P < 0.001; **Figure 1D**). Interestingly, serum GDF-15 was related to some characteristics, including SLEDAI (r_s_ = 0.502, P < 0.001), ESR (r_s_ = 0.303, P = 0.032), C3 (r_s_ = −0.391, P = 0.020), and C4 (r_s_ = −0.297, P = 0.035) (**Figures 1E–H**).

**Table 1:** Characteristics of SLE patients and healthy controls.

Characteristics	SLE patients	Healthy controls	P
Male (%)/female (%)	11.48/88.52	7.05/92.95	0.033
Age (year)	44.00 (27.00–52.00)	38.0 (31.00–48.00)	0.931
ESR (mm/H)	33.00 (13.00–55.00)	–	–
CRP (mg/L)	1.50 (0.20–17.25)	–	–
IgG (g/L)	14.05 (11.15–19.20)	–	–
IgA (mg/L)	3.03 (2.16–3.78)	–	–
IgM (mg/L)	0.94 (0.68–1.42)	–	–
C3 (g/L)	0.787 (0.513–0.992)	–	–
C4 (g/L)	0.178 (0.084–0.279)	–	–
RF (IU/mL)	9.60 (7.40–13.50)	–	–
SLEDAI	11.00 (5.50–18.00)	–	–
Arthritis, n (%)	180 (41.57)	–	–
Discoid rash, n (%)	162 (37.41)	–	–
Alopecia, n (%)	123 (28.41)	–	–
Oral ulcers, n (%)	58 (13.39)	–	–
Vasculitis, n (%)	36 (8.31)	–	–
Pleurisy, n (%)	32 (7.39)	–	–
Pericarditis, n (%)	34 (7.85)	–	–
Fever, n (%)	81 (18.71)	–	–
Hypocomplementemia, n (%)	208 (48.04)	–	–
Anti-dsDNA (+), n (%)	101 (23.33)	–	–
Thrombocytopenia, n (%)	71 (16.40)	–	–
Leukopenia, n (%)	47 (10.85)	–	–
Hematuria, n (%)	140 (32.33)	–	–
Proteinuria, n (%)	210 (48.50)	–	–
Pyuria, n (%)	42 (9.70)	–	–
ANA (+), n (%)	230 (53.12)	–	–
Anti-Sm (+), n (%)	109 (25.17)	–	–
Anti-SSA (+), n (%)	183 (42.26)	–	–
Anti-SSB (+), n (%)	57 (13.16)	–	–
Anti-RNP (+), n (%)	139 (32.10)	–	–

SLE, systemic lupus erythematosus; ESR, erythrocyte sedimentation rate; CRP, C-reactive protein; RF, rheumatoid factor; SLEDAI, SLE disease activity index.

To better evaluate the capacity of serum GDF-15 in distinguishing SLE from healthy controls, the diagnostic efficiency of serum GDF-15 was calculated (**Table 2**). Compared with healthy controls, the sensitivity, specificity, +LR, −LR, Youden’s index, accuracy, PPV, and NPV in SLE patients were 0.907, 0.800, 4.537, 0.116, 0.707, 0.840, 0.731, and 0.935, respectively, at cutoff value 230.993 pg/ml. Thus, GDF-15 was related to SLE pathogenesis and may be a potential biomarker to distinguish SLE patients from healthy controls.

**Table 2:** Capacity of serum GDF-15 in distinguishing SLE from healthy controls.

Comparison	GDF-15^*^	SLE	HC	Sensitivity	Specificity	+LR	−LR	Youden’s index	Accuracy	PPV	NPV	Cutoff value (pg/ml)
SLE vs. HC	+	49	18	0.907	0.800	4.537	0.116	0.707	0.840	0.731	0.935	230.993
	−	5	72									

^*^+, individual serum level of GDF-15 is higher than the cut-off value. −, individual serum level of GDF-15 is lower than the cutoff value.

SLE, systematic lupus erythematosus; HC, healthy controls; LR, likelihood ratio; PPV, positive predictive value; NPV, negative predictive value.

### GDF-15 Gene Polymorphisms Relate to SLE Risk

Seven polymorphisms were detected from 289 SLE patients and 525 healthy controls. Hardy–Weinberg equilibrium test for the seven polymorphisms was conducted (**Supplementary Table 3**). The powers were 0.987 for rs1055150, 0.968 for rs1058587, 0.978 for rs1059369, 0.980 for rs1059519, 0.819 for rs1227731, 0.983 for rs4808793, and 0.968 for rs16982345 to detect a 1.85-fold increased risk assuming an α value of 0.05. Because sex between SLE patients and healthy controls was not matched, association of the polymorphisms between SLE patients and controls was adjusted (**Table 3**). Frequencies of genotypes CC and GC of rs1055150 in SLE patients were higher than those in controls [CC vs. GG, OR (95% CI): 2.123 (1.178–3.828), P = 0.012; GC vs. GG, OR (95% CI): 2.675 (1.485–4.818), P = 0.001]. In a recessive model (GG vs. GC+CC), frequency of genotype GG of rs1055150 was lower in SLE patients (OR (95% CI): 0.420 (0.238–0.742), P = 0.003). For rs1059369, increased frequencies of TT + AT genotypes and decreased frequency of allele A were found in SLE patients [TT + AT vs. AA (dominant model), OR (95% CI): 1.454 (1.069–1.977), P = 0.017; A vs. T, OR (95% CI): 0.803 (0.653–0.986), P = 0.036]. Frequencies of GG, GC, and GG + GC genotypes for rs1059519 were higher in SLE patients when compared with those in healthy controls [GG vs. CC, OR (95% CI): 2.059 (1.141–3.715), P = 0.016; GC vs. CC, OR (95% CI): 2.729 (1.512–4.926), P = 0.001; CC vs. (GG + GC) (recessive model), OR (95% CI): 0.423 (0.239–0.749), P = 0.003]. Compared with healthy controls, frequencies of CC and CG genotypes for rs4808793 in SLE patients were elevated, and in a recessive model, frequencies of GG genotype were declined in SLE patients [CC vs. GG, OR (95% CI): 1.796 (1.004–3.210), P = 0.048; CG vs. GG, OR (95% CI): 2.414 (1.351–4.311), P = 0.003; GG vs. (CC + CG) (recessive model), OR (95% CI): 0.481 (0.275, 0.842), P = 0.010]. There was no significant difference regarding allele or genotype distribution between SLE patients and controls for rs1058587, rs1227731, and rs16982345.

**Table 3:** Allele and genotype frequencies of seven GDF-15 gene polymorphisms between SLE patients and healthy controls.

Polymorphism	Model	SLE, n (%)	Controls, n (%)	Before adjustment		After adjustment^a^	
				OR (95% CI)	P-value	OR (95% CI)	P-value
rs1055150	Genotypes						
	CC	129 (44.6)	245 (46.7)	2.106 (1.170–3.791)	0.013	2.123 (1.178–3.828)	0.012
	GC	144 (49.8)	216 (41.1)	2.667 (1.483–4.796)	0.001	2.675 (1.485–4.818)	0.001
	GG	16 (5.6)	64 (12.2)	Reference		Reference	
	Allele						
	C	402 (69.6)	706 (67.2)	1.113 (0.894–1.386)	0.338	–	–
	G	176 (30.4)	344 (32.8)	Reference			
	Dominant model						
	GG+GC	160 (55.4)	280 (53.3)	1.085 (0.813–1.448)	0.578	1.079 (0.808–1.441)	0.605
	CC	129 (44.6)	245 (46.7)	Reference		Reference	
	Recessive model						
	GG	16 (5.5)	64 (12.2)	0.422 (0.239–0.745)	0.002	0.420 (0.238–0.742)	0.003
	GC+CC	273 (94.5)	461 (87.8)	Reference		Reference	
rs1058587	Genotypes						
	GG	151 (52.2)	259 (49.3)	1.166 (0.640–2.125)	0.616	1.155 (0.633–2.108)	0.639
	GC	120 (41.5)	230 (43.8)	1.043 (0.568–1.915)	0.891	1.040 (0.566–1.912)	0.899
	CC	18 (6.22)	36 (6.9)	Reference		Reference	
	Allele						
	G	422 (73.0)	748 (71.2)	0.916 (0.730–1.149)	0.447	–	–
	C	156 (27.0)	302 (28.8)	Reference			
	Dominant model						
	CC+GC	138 (47.8)	266 (50.7)	0.890 (0.668–1.186)	0.426	0.896 (0.672–1.195)	0.896
	GG	151 (52.2)	259 (49.3)	Reference		Reference	
	Recessive model						
	CC	18 (6.2)	36 (6.9)	0.902 (0.503–1.619)	0.730	0.908 (0.505–1.633)	0.748
	GG+GC	271 (93.8)	489 (93.1)	Reference		Reference	
rs1059369	Genotypes						
	AA	87 (30.1)	203 (38. 7)	0.690 (0.453–1.053)	0.086	0.695 (0.455–1.062)	0.093
	AT	148 (51.2)	235 (44.8)	1.015 (0.682–1.509)	0.943	1.015 (0.682–1.512)	0.941
	TT	54 (18.7)	87 (16.6)	Reference		Reference	
	Allele						
	A	322 (55.7)	641 (61.0)	0.803 (0.653–0.986)	0.036	–	–
	T	256 (44.3)	409 (39.0)	Reference			
	Dominant model						
	TT+AT	202 (69.9)	322 (61.3)	1.464 (1.077–1.989)	0.015	1.454 (1.069–1.977)	0.017
	AA	87 (30.1)	203 (38.7)	Reference		Reference	
	Recessive model						
	TT	54 (18.7)	87 (16.6)	1.157 (0.795–1.683)	0.446	1.153 (0.792–1.679)	0.458
	AA+AT	235 (81.3)	438 (83.4)	Reference		Reference	
rs1059519	Genotypes						
	GG	130 (50.0)	252 (48.0)	2.031 (1.128–3.657)	0.018	2.059 (1.141–3.715)	0.016
	GC	143 (49.5)	210 (40.0)	2.681 (1.489–4.829)	0.001	2.729 (1.512–4.926)	0.001
	CC	16 (5.50)	63 (12.0)	Reference		Reference	
	Allele						
	G	403 (69.7)	714 (68.0)	0.923 (0.741–1.150)	0.473	–	–
	C	175 (30.3)	336 (32.0)	Reference			
	Dominant model						
	CC+GC	159 (55.0)	273 (52.0)	1.129 (0.846–1.506)	0.409	1.130 (0.847–1.509)	0.406
	GG	130 (45.0)	252 (48.0)	Reference		Reference	
	Recessive model						
	CC	16 (5.5)	63 (12.0)	0.430 (0.243–0.759)	0.003	0.423 (0.239–0.749)	0.003
	GG+GC	273 (94.5)	462 (88.0)	Reference		Reference	
rs1227731	Genotypes						
	GG	192 (66.4)	360 (68.6)	1.751 (0.632–4.852)	0.281	1.825 (0.655–5.080)	0.250
	GA	92 (31.8)	149 (28.4)	1.868 (0.661–5.278)	0.238	1.927 (0.679–5.469)	0.218
	AA	5 (1.80)	16 (3.0)	Reference		Reference	
	Allele						
	G	481 (83.2)	869 (82.8)	0.968 (0.739–1.269)	0.815	–	–
	A	97 (16.8)	181 (17.2)	Reference			
	Dominant model						
	AA+GA	92 (31.8)	165 (31.4)	1.019 (0.748–1.387)	0.095	1.006 (0.738–1.372)	0.967
	GG	197 (68.2)	360 (68.6)	Reference		Reference	
	Recessive model						
	AA	5 (1.7)	16 (3.0)	0.560 (0.203–1.545)	0.257	0.539 (0.194–1.494)	0.235
	GG+GA	284 (98.3)	509 (97.0)	Reference		Reference	
rs4808793	Genotypes						
	CC	127 (43.95)	251 (47.8)	1.786 (1.001–3.187)	0.050	1.796 (1.004–3.210)	0.048
	CG	145 (50.17)	214 (40.8)	2.391 (1.341–4.264)	0.003	2.414 (1.351–4.311)	0.003
	GG	17 (5.88)	60 (11.4)	Reference		Reference	
	Allele						
	C	399 (69.0)	716 (68.2)	1.040 (0.835–1.294)	0.727	–	–
	G	179 (31.0)	334 (31.8)	Reference			
	Dominant model						
	CG+GG	162 (56.1)	274 (52.2)	1.169 (0.876–1.559)	0.290	1.171 (0.877–1.564)	0.285
	CC	127 (43.9)	251 (47.8)	Reference		Reference	
	Recessive model						
	GG	17 (5.9)	60 (11.4)	0.484 (0.277–0.847)	0.010	0.481 (0.275–0.842)	0.010
	CC+CG	272 (94.1)	465 (88.6)	Reference		Reference	
rs16982345	Genotypes						
	GG	143 (49.48)	269 (51.2)	0.669 (0.400–1.117)	0.125	0.656 (0.392–1.098)	0.109
	GA	115 (39.79)	217 (41.3)	0.667 (0.395–1.125)	0.129	0.666 (0.392–1.125)	0.129
	AA	31 (10.73)	39 (7.5)	Reference		Reference	
	Allele						
	G	401 (69.4)	755 (71.9)	1.130 (0.905–1.411)	0.282	–	–
	A	177 (30.6)	295 (28.1)	Reference			
	Dominant model						
	GA+AA	146 (50.5)	256 (48.8)	1.073 (0.805–1.430)	0.631	1.093 (0.819–1.459)	0.545
	GG	143 (49.5)	269 (51.2)	Reference		Reference	
	Recessive model						
	AA	31 (10.7)	39 (7.4)	1.497 (0.913–2.457)	0.108	1.514 (0.921–2.488)	0.102
	GG+GA	258 (89.3)	486 (92.6)	Reference		Reference	

SLE, systemic lupus erythematosus.

a Adjustment by sex.

Association between the seven polymorphisms and clinical and laboratory parameters of SLE patients, such as SLEDAI, C3, C4, ESR, RF, IgA, IgM, IgG, CRP, ANA, anti-SSA, anti-SSB, was discussed in **Table 4**, **Supplementary Table 4**. SLEDAI was significantly different in patients with rs1227731 GG, GA, and AA genotypes [GG vs. GA vs. AA: 10.000 (4.000–14.000) vs. 9.000 (7.000–17.000) vs. 8.000 (6.000–8.500), P = 0.037]. Serum levels of complement C4 were different in patients for rs1227731 genotypes, and patients carrying AA genotype had lower serum C4 [GG vs. GA vs. AA, P = 0.008; (GG + GA) vs. AA, P = 0.004]. Serum levels of IgG were different in patients for rs1227731 (AA vs. AT vs. TT, P = 0.026), and SLE patients with TT genotype had lower serum levels of IgG (AA + AT vs. TT, P = 0.048). There was a higher frequency of allele G of rs1227731 in patients with anti-SSA (+) and anti-SSB (+) (P = 0.037, P = 0.038). For rs1059369, serum levels of IgA were reduced in patients with TT genotype (AA + AT vs. TT, P = 0.042). SLE patients with fever, anti-SSA (+), and anti-SSB (+) showed significant differences of frequencies of rs1059369 genotypes, compared with patients without these clinical features (P = 0.047, P = 0.033, P = 0.018). There was a higher frequency of allele A of rs1059369 in patients with fever compared with the patients without fever (P = 0.016). A lower frequency of allele A of rs1059369 in SLE patients with anti-SSA (+) was noted (P = 0.011). For rs16982345, patients with GG, GA, and AA genotypes had different levels of RF (P = 0.021), and there were increased levels of RF in patients with GG genotype (GG vs. GA+AA, P = 0.009). There was a higher frequency of allele G of rs16982345 in SLE patients with anti-dsDNA (+) (P = 0.024) and anti-RNP (+) (P = 0.026). There was a higher frequency of allele C of rs4808793 in patients with anti-SSB (+), and a lower frequency of allele C of rs4808793 in patients with pleurisy compared with those in patients without the features (P = 0.043, P = 0.045). There was a lower frequency of allele G of rs1059519 in patients with pleurisy (P = 0.034).

**Table 4:** Association of GDF-15 gene polymorphisms in SLE patients with clinical, laboratory features (quantitative variables).

Polymorphism	Characteristics	Wild-type genotypes	Heterozygosity	Mutant-type genotypes	P_1_	P_2_	P_3_
rs1055150		CC	CG	GG			
	SLEDAI	10.000 (4.000–16.500)	9.000 (4.250–15.500)	8.000 (6.000–12.000)	0.982	0.909	0.858
	C3	0.856 (0.565–1.048)	0.795 (0.541–0.960)	0.974 (0.907–1.253)	0.332	0.628	0.191
	C4	0.176 (0.113–0.285)	0.177 (0.083–0.273)	0.277 (0.120–0.401)	0.280	0.810	0.121
	ESR	31.500 (9.750–45.000)	16.500 (8.000–45.000)	19.000 (11.000–55.000)	0.397	0.182	0.699
	RF	10.150 (7.000–12.375)	9.500 (7.975–13.075)	9.000 (7.100–14.000)	0.667	0.395	0.898
	IgA	2.895 (1.778–4.055)	3.280 (2.490–3.740)	2.230 (1.400–2.820)	0.569	0.373	0.401
	IgM	0.755 (0.538–1.160)	1.015 (0.648–1.435)	1.230 (0.800–1.960)	0.737	0.539	0.731
	IgG	12.680 (10.995–15.115)	14.475 (11.070–18.860)	13.970 (7.880–18.640)	0.425	0.217	0.466
	CRP	4.250 (0.450–23.625)	1.090 (0.200–10.683)	1.200 (0.400–5.300)	0.755	0.523	0.584
rs1058587		GG	GC	CC			
	SLEDAI	8.000 (5.500–14.000)	12.000 (4.500–19.000)	8.000 (6.000–9.000)	0.834	0.293	0.275
	C3	0.801 (0.575–0.988)	0.824 (0.560–1.035)	1.195 (1.056–1.198)	0.207	0.725	0.571
	C4	0.175 (0.086–0.269)	0.240 (0.127–0.317)	0.178 (0.170–0.229)	0.858	0.780	0.701
	ESR	28.000 (9.000–58.500)	22.000 (8.500–35.000)	24.000 (18.000–27.500)	0.553	0.940	0.287
	RF	9.600 (8.150–15.100)	10.000 (6.850–11.350)	8.200 (4.100–8.950)	0.827	0.795	0.546
	IgA	2.820 (2.140–3.745)	2.830 (1.985–3.630)	3.720 (3.500–3.820)	0.433	0.202	0.905
	IgM	0.870 (0.625–1.490)	0.940 (0.680–1.385)	0.910 (0.800–0.940)	0.732	0.729	0.557
	IgG	13.970 (11.500–18.795)	13.270 (11.075–17.115)	12.750 (11.640–13.025)	0.655	0.909	0.362
	CRP	1.200 (0.200–11.900)	1.900 (0.400–16.700)	11.900 (6.100–15.750)	0.148	0.285	0.222
rs1059369		AA	AT	TT			
	SLEDAI	9.000 (4.500–13.000)	9.500 (4.750–17.250)	9.500 (3.250–16.000)	0.523	0.795	0.732
	C3	0.940 (0.815–1.053)	0.749 (0.492–0.911)	0.919 (0.565–1.024)	0.875	0.262	0.867
	C4	0.243 (0.135–0.309)	0.172 (0.088–0.264)	0.155 (0.091–0.285)	0.784	0.564	0.581
	ESR	22.000 (11.500–38.000)	24.500 (8.000–42.750)	37.500 (8.500–82.500)	0.977	0.898	0.837
	RF	9.400 (6.900–11.350)	9.400 (7.225–18.200)	10.900 (8.500–13.125)	0.830	0.685	0.793
	IgA	3.150 (2.195–3.615)	2.950 (2.395–3.943)	2.310 (1.620–4.058)	0.126	0.501	0.042
	IgM	0.970 (0.760–1.425)	0.875 (0.598–1.500)	0.705 (0.448–0.973)	0.505	0.627	0.244
	IgG	13.630 (10.770–17.860)	14.475 (11.370–19.035)	12.115 (8.995–14.358)	0.026	0.253	0.048
	CRP	3.970 (0.300–19.450)	0.640 (0.200–6.175)	8.700 (0.425–20.400)	0.352	0.467	0.337
rs1059519		GG	GC	CC			
	SLEDAI	10.000 (4.750–16.000)	8.500 (4.000–14.750)	8.000 (6.000–12.000)	0.913	0.627	0.791
	C3	0.914 (0.577–1.057)	0.782 (0.526–0.925)	0.974 (0.907–1.253)	0.171	0.991	0.121
	C4	0.176 (0.116–0.279)	0.177 (0.078–0.264)	0.277 (0.120–0.401)	0.118	0.943	0.066
	ESR	31.000 (9.250–42.250)	20.000 (8.000–49.000)	19.000 (11.000–55.000)	0.561	0.462	0.727
	RF	10.150 (7.225–12.925)	9.500 (7.675–12.225)	9.000 (7.100–14.000)	0.723	0.809	0.843
	IgA	2.895 (1.713–3.988)	3.280 (2.530–3.795)	2.230 (1.400–2.820)	0.846	0.485	0.867
	IgM	0.730 (0.513–1.113)	1.070 (0.663–1.453)	1.230 (0.800–1.960)	0.600	0.169	0.943
	IgG	12.415 (10.648–14.778)	14.860 (11.813–19.598)	13.970 (7.880–18.640)	0.840	0.101	0.698
	CRP	3.750 (0.350–18.500)	1.090 (0.200–12.925)	1.200 (0.400–5.300)	0.881	0.463	0.935
rs1227731		GG	GA	AA			
	SLEDAI	10.000 (4.000–14.000)	9.000 (7.000–17.000)	8.000 (6.000–8.500)	0.037	0.649	0.292
	C3	0.824 (0.577–1.020)	0.780 (0.515–0.975)	0.974 (0.941–1.114)	0.458	0.028	0.080
	C4	0.178 (0.116–0.260)	0.170 (0.058–0.278)	0.288 (0.283–0.345)	0.008	0.082	0.004
	ESR	28.000 (8.000–43.000)	22.000 (9.000–55.000)	14.000 (12.500–18.000)	0.286	0.118	0.563
	RF	10.200 (8.000–12.100)	9.000 (7.100–13.500)	8.400 (5.950–8.700)	0.428	0.945	0.223
	IgA	3.170 (1.930–3.930)	2.830 (2.230–3.460)	2.820 (2.110–2.985)	0.545	0.313	0.828
	IgM	0.800 (0.590–1.190)	1.180 (0.880–1.830)	0.850 (0.785–1.040)	0.577	0.845	0.344
	IgG	12.750 (10.560–15.370)	16.400 (13.970–18.870)	11.850 (9.490–15.245)	0.557	0.497	0.333
	CRP	3.700 (0.300–19.600)	0.400 (0.200–3.970)	1.200 (0.950–3.250)	0.959	0.868	0.848
rs4808793		CC	CG	GG			
	SLEDAI	10.000 (4.000–16.000)	9.000 (4.000–14.000)	8.000 (6.000–12.000)	0.481	0.823	0.895
	C3	0.911 (0.572–1.031)	0.784 (0.529–0.971)	0.974 (0.907–1.253)	0.959	0.553	0.245
	C4	0.173 (0.116–0.279)	0.178 (0.081–0.277)	0.277 (0.120–0.401)	0.351	0.666	0.149
	ESR	31.000 (9.000–43.000)	18.000 (8.000–46.000)	19.000 (11.000–55.000)	0.723	0.431	0.980
	RF	10.000 (7.000–12.100)	9.600 (7.800–13.500)	9.000 (7.100–14.000)	0.521	0.256	0.843
	IgA	2.760 (1.680–4.010)	3.300 (2.550–3.750)	2.230 (1.400–2.820)	0.726	0.631	0.617
	IgM	0.730 (0.500–1.160)	1.060 (0.670–1.450)	1.230 (0.800–1.960)	0.346	0.145	0.713
	IgG	12.680 (10.530–15.030)	14.730 (11.280–18.870)	13.970 (7.880–18.640)	0.326	0.200	0.666
	CRP	4.400 (0.500–19.600)	0.680 (0.200–10.800)	1.200 (0.400–5.300)	0.668	0.414	0.868
rs16982345		GG	GA	AA			
	SLEDAI	8.500 (4.750–12.250)	9.000 (4.000–22.000)	11.500 (7.250–15.500)	0.702	0.811	0.766
	C3	0.895 (0.568–1.010)	0.784 (0.572–1.021)	0.914 (0.589–1.128)	0.896	0.751	0.536
	C4	0.181 (0.089–0.338)	0.178 (0.116–0.278)	0.144 (0.073–0.222)	0.730	0.635	0.446
	ESR	22.000 (8.750–58.250)	28.000 (8.000–36.000)	21.000 (9.750–34.000)	0.947	0.961	0.775
	RF	11.000 (8.875–16.650)	9.200 (5.600–10.900)	8.450 (2.800–9.550)	0.021	0.009	0.088
	IgA	2.755 (2.103–3.690)	3.030 (2.160–3.930)	3.500 (1.865–3.878)	0.181	0.065	0.616
	IgM	0.955 (0.730–1.533)	0.850 (0.600–1.380)	0.675 (0.475–0.955)	0.699	0.446	0.951
	IgG	13.245 (10.473–18.840)	13.630 (11.190–17.460)	13.650 (11.085–18.873)	0.454	0.439	0.511
	CRP	1.350 (0.200–10.448)	5.400 (0.500–24.390)	2.540 (0.300–17.675)	0.894	0.829	0.637

SLEDAI, systemic lupus erythematosus disease activity index; ESR, erythrocyte sedimentation rate; CRP, C-reactive protein; RF, rheumatoid factor.

^1^Wild-type genotypes vs. Heterozygosity vs. Mutant-type genotypes.

^2^Wild-type genotypes vs. (heterozygosity + mutant-type genotypes).

^3^(Wild-type genotypes + heterozygosity) vs. mutant-type genotypes.

**Figure 2:**
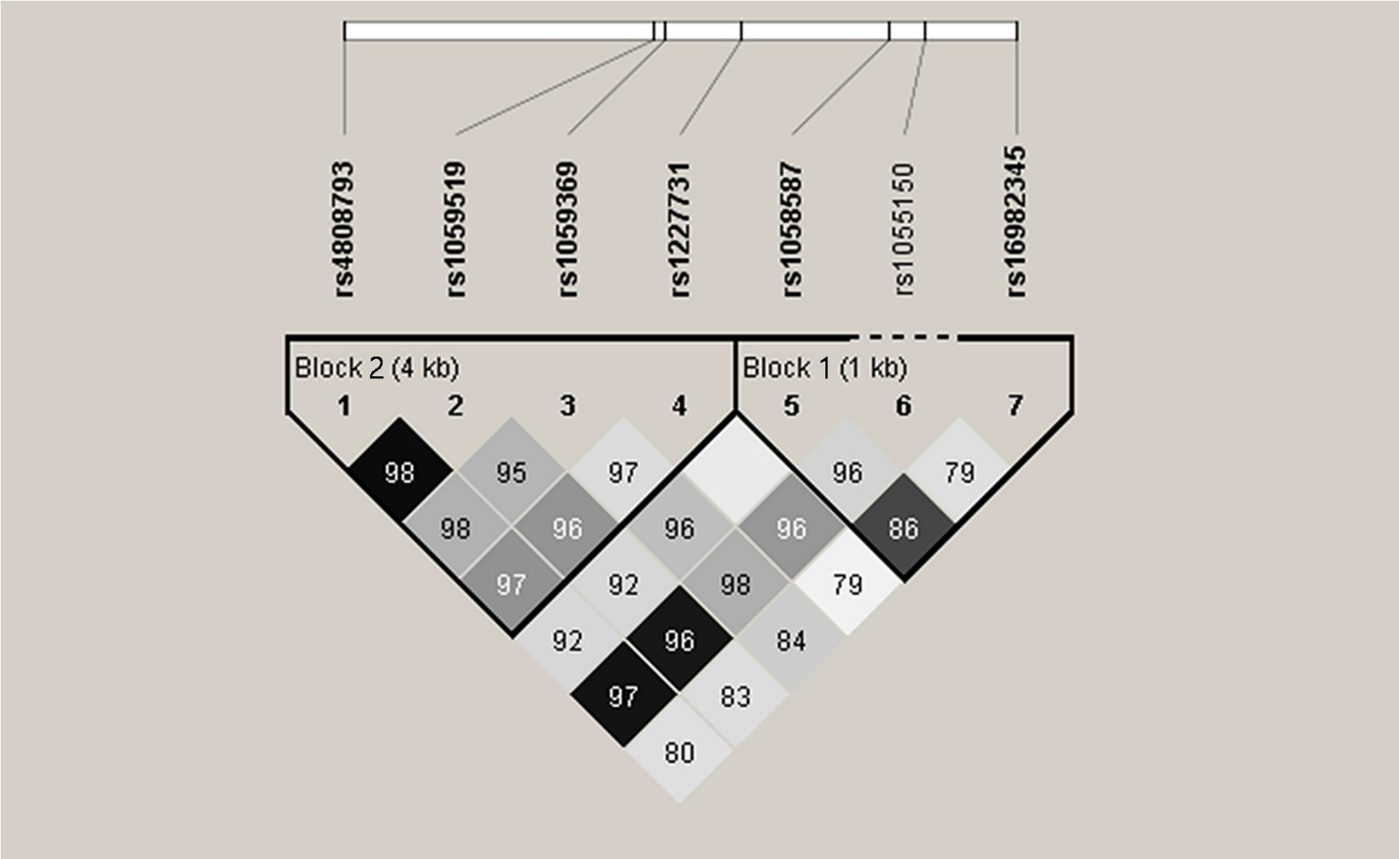
Linkage disequilibrium (LD) of seven single-nucleotide polymorphisms in GDF-15 gene. Intensity of linkage disequilibrium (LD) is reflected in each box by color and numeric value. The number is disequilibrium value (D′) (for example, 98 means D′ = 0.98). Correlation degree of two crossed polymorphisms is reflected by color shading: Black means r^2^ > 0.8; dark gray means r^2^ from 0.4 to 0.8; light gray means r^2^ < 0.4. There are two blocks. Block 1 consists of rs16982345 and rs1058587. Block 2 consists of rs4808793, rs1059519, rs1059369, and rs1227731.

Haplotype analysis was further explored with respect to association of GDF-15 haplotypes with SLE risk. Two blocks were defined. Block 1 consists of rs16982345 and rs1058587, and block 2 consists of rs4808793, rs1059519, rs1059369, and rs1227731 (**Figure 2**). Results showed that higher frequencies of GG and CA haplotypes and lower frequencies of GA and CG haplotypes were noted in SLE patients than those in healthy controls (All P < 0.005). Frequency of CGTG haplotype was lower in SLE patients compared with that in controls (P = 0.034, **Table 5**).

**Table 5:** Haplotype analysis for GDF-15 gene polymorphisms between SLE patients and healthy controls.

Blocks^*^	Haplotype	Frequency	SLE ratio	Control ratio	χ^2^	P
1	GG	0.684	0.708	0.639	8.237	0.004
	CA	0.255	0.277	0.215	7.433	0.006
	GA	0.035	0.004	0.091	83.784	<0.001
	CG	0.026	0.011	0.054	28.121	<0.001
2	CGTG	0.400	0.381	0.435	4.474	0.034
	CGAG	0.279	0.293	0.252	3.109	0.078
	GCAA	0.165	0.167	0.160	0.151	0.697
	GCAG	0.144	0.147	0.138	0.244	0.621

^*^Block 1 consists of rs16982345 and rs1058587. Block 2 consists of rs4808793, rs1059519, rs1059369, and rs1227731. SLE, systemic lupus erythematosus. SLE patient versus controls by 2×2 chi-square test.

### GDF-15 Treatment Did Not Relieve Lupus Mice Hepatomegaly and Splenomegaly

**Figure 3:**
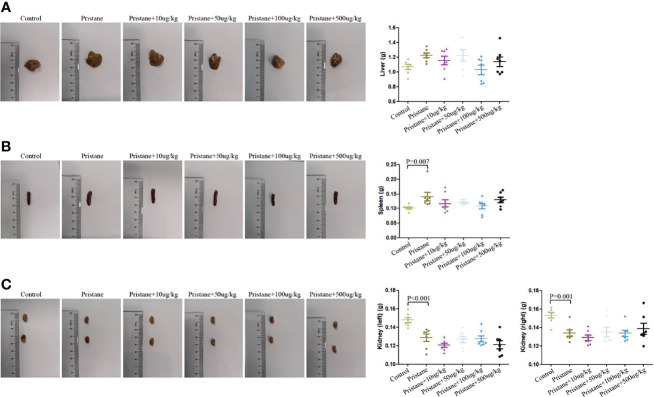
Hepatomegaly and splenomegaly were not relieved by GDF-15 treatment. **(A–C)** Weight of the individual liver, spleen, and kidneys (left and right) and representative photographs in six groups, including WT mice (injection with phosphate-buffered saline (PBS) in all experimental period), pristane-induced lupus mice with PBS injection, and pristane-induced lupus mice injected with 10, or 50, or 100, or 500 μg/kg GDF-15. Seven samples per group were analyzed by Student’s t-test. Comparison is conducted among pristane-induced lupus mice with PBS injection and other five groups. P-value < 0.01 was significant under Bonferroni correction.

As shown in **Figure 3**, reduced weight of both kidneys was observed after pristane induction in WT mice, and the weight of spleen was increased in WT mice treated with pristane (all P < 0.01). GDF-15 treatment did not significantly reduce the weight of the liver, spleen, and kidneys in lupus mice (**Figures 3A–C**).

### Alleviated Renal Damage After GDF-15 Treatment in Lupus Mice

**Figure 4:**
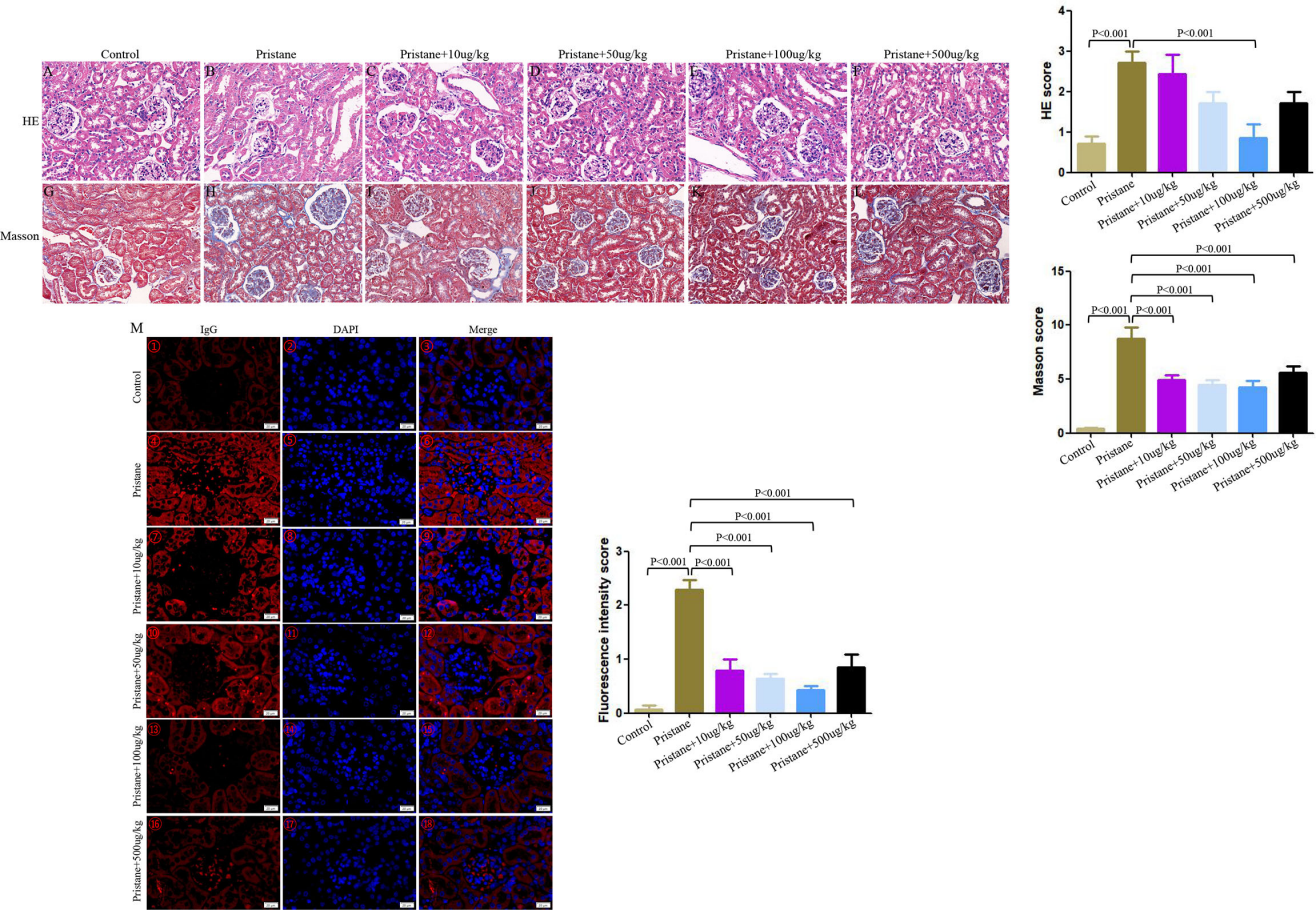
GDF-15 treatment alleviated renal damage of lupus mice. Photomicrographic representation of renal damage. **(A–F)** Hematoxylin and eosin (HE), **(G–L)** Masson, and **(M)** Immunofluorescence assay in six groups, including wild-type mice (injection with PBS in all experimental period), pristane-induced lupus mice with PBS injection, and pristane-induced lupus mice injected with 10, or 50, or 100, or 500 μg/kg GDF-15. Magnification ×400 for HE, Masson assay, immunofluorescence assay. HE and Masson scores are means ± standard deviation (SD). The fluorescence intensity scores for kidneys with symbols in right panels (M①-⑱) are means ± SD. Comparison is conducted among pristane-induced lupus mice with PBS injection and other five groups. P-value < 0.01 was significant under Bonferroni correction.

In pristane-induced lupus mice, glomerular atrophy and necrosis, mesangial proliferation, basement membrane thickening, capillary shrinkage, endothelial cell nuclear enlargement, renal tubular degeneration, lymphocyte infiltration, and fibrous tissue hyperplasia were observed. Similarly, the lupus mice had severe IgG deposition compared with wild-type (WT) mice. However, renal damage and IgG deposition were alleviated when lupus mice were treated with GDF-15. Scores of HE, Masson, and fluorescence intensity indicated that lupus mice injected with 100 μg/kg GDF-15 showed the highest therapeutic efficacy (**Figures 4A–M**).

### GDF-15 Treatment Reversed Immune Cells Dysregulation

**Figure 5:**
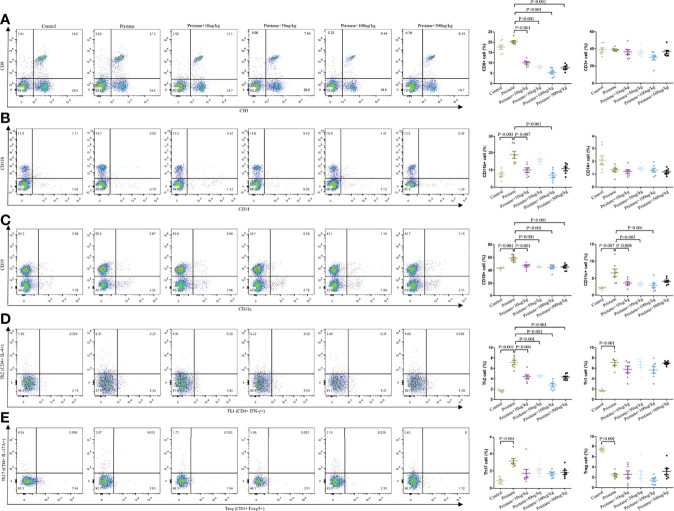
GDF-15 treatment reversed immune cells dysregulation. Flow cytometry analysis for different immune cells in six groups, including wild-type mice (injection with PBS in all experimental period), pristane-induced lupus mice with PBS injection, and pristane-induced lupus mice injected with 10, or 50, or 100, or 500 μg/kg GDF-15. Percentages of CD8^+^, CD3^+^, CD11b^+^, CD14^+^, CD19^+^, CD11c^+^, CD4^+^IFNγ^+^, CD4^+^IL-4^+^, CD4^+^IL-17A^+^, and CD4^+^Foxp3^+^ cells in six groups of mice (left panel). **(A–E)** right panel: Symbols represent individual mice. Seven samples per group were analyzed by Student’s t-test. Comparison is conducted among pristane-induced lupus mice with PBS injection and other five groups. P-value < 0.01 was significant under Bonferroni correction.

Higher frequencies of CD11b^+^, CD19^+^, CD11C^+^, T_H_2, T_H_1, and T_H_17 cells and lower frequencies of T_reg_ cells were observed in pristane-induced lupus mice than those in WT mice (**Figures 5A–E**). After GDF-15 treatment (100 µg/kg), CD8^+^, CD11b^+^, CD19^+^, CD11C^+^, and T_H_2 cells were significantly decreased compared to those in pristane-induced lupus mice, and this was the most efficient compared to what other does (**Figures 5A–D**).

### Reduced Inflammatory Cytokines and Autoantibodies Production in GDF-15–Treated Lupus Mice

**Figure 6:**
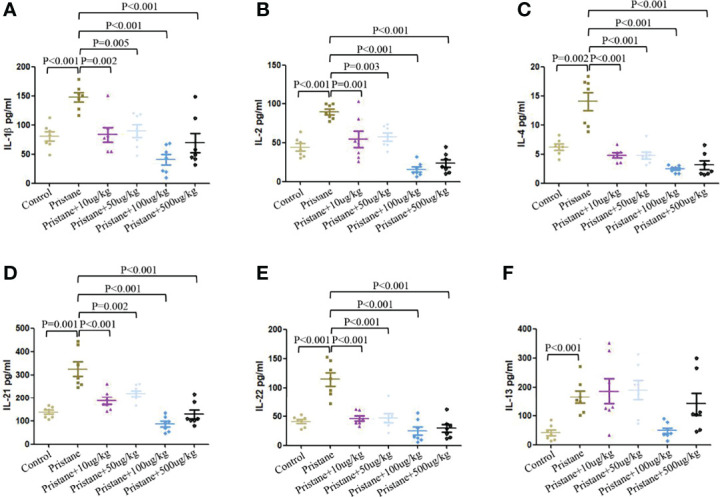
GDF-15 treatment reduced serum inflammatory cytokines secretion. **(A–F)** Serum levels of inflammatory cytokines from six groups were evaluated by microarray, including wild-type mice (injection with PBS in all experimental period), pristane-induced lupus mice with PBS injection, and pristane-induced lupus mice injected with 10, or 50, or 100, or 500 μg/kg GDF-15. Cytokines included IL-1β, IL-2, IL-4, IL-13, IL-21, and IL-22. Symbols represent individual mice. Bars show the means ± SD. A total of seven samples per group were analyzed by Student’s t-test. Comparison is conducted among pristane-induced lupus mice with PBS injection and other five groups. P-value < 0.01 was significant under Bonferroni correction.

As shown in **Figure 6**, pristane-induced lupus mice showed significantly higher serum levels of IL-1β, IL-2, IL-4, IL-13, IL-21, and IL-22 compared to those in WT mice (**Figures 6A–F**). GDF-15 treatment significantly reduced levels of IL-1β, IL-2, IL-4, IL-21, and IL-22 (**Figures 6A–E**), by which treatment of lupus mice with 100 µg/kg GDF-15 had the most efficiency. Some cytokines (IL-5, IL-6, IL-12p70, IL-17A, IL-28A, IL-17F, IFNγ, MIP-3α, TGF-β1, and TNF-α) were not detectable in some groups of mice (data not shown).

**Figure 7:**
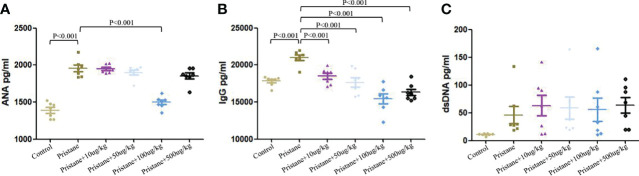
GDF-15 treatment reduced autoantibodies production. **(A-C)** Serum levels of anti-nuclear antibody (ANA), total IgG, and anti-dsDNA were examined in six groups, including wild-type mice (injection with PBS in all experimental period), pristane-induced lupus mice with PBS injection, and pristane-induced lupus mice injected with 10, or 50, or 100, or 500 μg/kg GDF-15. Symbols represent individual mice. Bars show the means ± SD. A total of seven samples per group were analyzed by Student’s t-test. Comparison is conducted among pristane-induced lupus mice with PBS injection and other five groups. P-value < 0.01 was significant under Bonferroni correction.

Compared with WT mice, pristane-induced lupus mice showed significantly higher levels of ANA and total IgG (**Figures 7A, B**). Addition of GDF-15 treatment significantly reduced ANA and total IgG levels in lupus mice, by which 100 μg/kg GDF-15 injection had the best efficacy (**Figures 7A, B**). GDF-15 treatment did not significantly affect anti-dsDNA levels (**Figure 7C**).

## Discussion

In this study, two population-based case-control studies were conducted, which not only discussed the association of GDF-15 in SLE pathogenesis, the potential to be a marker for SLE, but also evaluated the GDF-15 genetic risk to SLE. Lupus mouse model further demonstrated the role of GDF-15 in lupus development.

Two studies (Greek and Chinese) with small sample sizes evaluated GDF-15 levels, which both showed higher expression of GDF-15 in SLE patients as compared to that in controls (12, 13). Our study confirmed again that serum levels of GDF-15 were much higher in SLE patients and positively correlated with hematuria, SLEDAI, and ESR, whereas negatively correlated with C3, C4. Chen et al. showed that SLE patients with active disease activity (SLEDAI > 4) had comparable GDF-15 expression with less active SLE patients and 24-h urine protein associated with GDF-15 expression (13). In our study, patients with high disease activity (SLEDAI ≥ 10) had increased levels GDF-15. These differences may relate to several reasons. The first is the source of SLE patients. Patients in our study were treatment naive, whereas patients had treatment in the other study. The second is that the criteria for active disease and less active disease are different. In our study, SLEDAI was evaluated according to the international standard. The third is that sample sizes are different. Moreover, patients with long disease duration may have chronic renal injury, such as the SLE patients recruited in the work of Chen et al. Thus, 24-h urine protein, as an indicator of chronic renal injury associated with GDF-15 expression, was reported in the work of Chen et al., but we showed that expression of GDF-15 was related to some indexes related acute renal injury, such as hematuria. Our study also found the relationship of ESR, C3, C4, and GDF-15 expression in SLE patients. All these indicated that GDF-15 may correlate with SLE pathogenesis. However, mechanisms that GDF-15 regulates the indexes to involve in lupus pathogenesis need further elucidation. Being a chronic disease, SLE is sometimes flared. Searching for potential markers to early detect SLE, or distinguish SLE from other non-SLE diseases may help to better, treats earlier SLE patients. In our study, we detected the ability of serum GDF-15 to distinguish SLE from healthy controls. Results showed good ability. Therefore, serum GDF-15 may be a biomarker to discriminate SLE patients from healthy individuals.

Genetic mutation may also be the marker for human beings. To discuss association of GDF-15 gene polymorphisms and SLE risk, a case-control study was firstly conducted in Chinese Han population. We found genotypes of rs1055150 (CC, CG), rs1059519 (GG, GC), rs1059369 (TT+AT), and rs4808793 (CC, CG) associated with SLE susceptibility and rs1059369, rs1059519, rs1227731, rs4808793, and rs16982345 associated with some clinical and laboratory characteristics of SLE patients. Patients with genotype CC or GC of rs1055150 had higher risk of SLE as compared to patients with GG genotype. Rs1055150 was located at 3’UTR. G to C mutation suggested higher risk of SLE, which may lead to increased GDF-15 mRNA expression, and increased serum levels of GDF-15 in SLE patients. Rs1059519 was located at exon, which has been reported to associate with GDF-15 expression and relate to chronic hepatitis C infection, left ventricular hypertrophy (20–22). In our study, rs1059519 polymorphism was related to SLE risk and SLE complicated with discoid and pleurisy, suggesting that rs1059519 may regulate vascular injury and inflammation. Rs1059369 was located at exon (a missense variant). A genome-wide association study showed association of rs1059369 with GDF-15 expression, multiple myeloma, hypertension, hepatitis C virus infection (20, 22). The present study found that SLE patients had higher frequencies of rs1059369 TT + AT genotypes and allele T than healthy controls, which were related to more serious disease activity, evidenced by lower IgA and IgG expression and higher expression of anti-SSA and anti-SSB in SLE patients. Rs4808793 and rs1227731 were located at intronic region, which may result in intron variants by changing transcript consequences. Then, the mutations may lead to disease pathogenesis. SLE patients with discoid or pleuritic had a higher frequency of CG genotype of rs4808793, and patients with anti-SSB (+) had a higher frequency of allele C of rs4808793 than those without these complications. For rs1227731, a lower frequency of GG genotype in SLE patients with fever and a higher frequency of allele G in SLE patients with anti-SSA (+) or anti-SSB (+) were observed. SLE patients with AA genotype of rs1227731 had higher expression of C4 than patients with AA or AT genotype. The findings indicated that GDF-15 genetic mutation correlated with SLE risk in Chinese Han population. However, how the mutations contribute to lupus pathogenesis needs to be discussed in the future, and discussing the potential of GDF-15 gene polymorphisms as genetic marker for SLE is necessary to be confirmed with multi-center, larger sample sizes.

Because GDF-15 correlated with SLE as discussed above, role of GDF-15 involved in lupus development was further demonstrated *in vivo*. Lupus mice treated with GDF-15 had alleviated renal damage. In pristane-induced lupus mice, there were higher frequencies of CD11b^+^, CD19^+^, CD11c^+^, T_H_1, T_H_2, and T_H_17 cells and lower frequencies of T_reg_ cells. A previous study showed that GDF-15 expression was increased in CD11b^+^ cells upon lipopolysaccharide (LPS)-induced inflammatory conditions (23). In the present study, GDF-15 treatment reversed the increased percentage of CD11b^+^ cells in lupus mice, suggesting that there may be a negative feedback mechanism that high expression of GDF-15 could restrain CD11b^+^ cells proliferation. Percentage of CD11c^+^ cells in white adipose tissue of obese mice was reduced after GDF-15 treatment (24). In patients with prediabetes, the number of senescent CD8^+^ T cells was positively related to serum levels of GDF-15, implying that increased GDF-15 may accelerate CD8^+^ T cells aging and further reduce number of CD8^+^ T cells (25). Consistently, our study showed that the proportion of CD11c^+^ and CD8^+^ cells was decreased when GDF-15 was injected into lupus mice. Moreover, percentages of CD19^+^, CD3^+^, and T_H_2 cells were reduced in lupus mice by GDF-15 treatment. Thus, GDF-15 is required for maintaining homeostasis, which relieves lupus progression by regulating innate and adaptive immune responses.

To date, the role of GDF-15 in regulating pro- or anti-inflammatory cytokines production is controversial. In GDF-15 gene–deficient mice, serum levels of IL-1β were reduced, which inhibited inflammation accumulation and development of atherogenesis (26). WT mice treated with LPS and GDF-15 had reduced expression of IL-1β, TNF-α, and IL-6 (27). Our findings showed that IL-1β expression was higher in pristane-induced lupus mice, and expression of IL-1β was significantly inhibited by GDF-15 treatment. IL-4 and IL-13, both induced by type 2 immune responses, were overexpressed in lupus mice. Recombinant IL-4 and IL-13 stimulation increased expression of GDF-15 in primary hepatocytes (28). In this study, GDF-15 treatment reduced percentage of T_H_2 cell and serological levels of IL-14. Thus, GDF-15 inhibited type 2 immune responses. Moreover, increased expression of IL-2, IL-21, and IL-22 in lupus mice was downregulated by GDF-15 treatment. Autoantibodies play momentous roles in SLE progression. In this study, we did not find significant relation of serum GDF-15 with ANA, ds-DNA, and total IgG in population-based study. However, GDF-15 treatment significantly reduced levels of ANA and total IgG in lupus mice. This confirmed the role of GDF-15 in inhibiting immune complex deposition in the kidney. The findings suggested that high expression of GDF-15 can inhibit production of autoantibodies and inflammatory cytokines. However, some studies reported that GDF-15 may activate inflammatory pathways, such as phosphorylation of extracellular signal–regulated kinase and RAC-alpha serine–threonine protein kinase (AKT) (22, 29).

In this study, we found that serum levels of GDF-15 were positively associated with SLEDAI. The pristane-induced lupus mice treated with GDF-15 had alleviated disease activity. Clarifying the divergence between disease suppressing effects of GDF-15 in the mouse model and its positive correlation with SLEDAI in SLE patients is useful to understand role of GDF-15 in lupus. However, the mechanism is not clear to date. Previous studies found that anti-inflammatory cytokine IL-37 levels were increased in RA, OA, AS, and SLE patients and were positively associated with RA (30), OA (31), AS (32), and SLE (33) disease activity. The increased IL-37 could reduce disease activity of the above diseases by inhibiting production of inflammatory cytokines (30–33), limiting T_H_17 cell proliferation (32), and enhancing the stability and effectiveness of mesenchymal stem cells (34), which were similar to our previous studies (35, 36). All the published studies showed that there may be a negative feedback mechanism between IL-37 and the above diseases, which has not been clearly elucidated (32, 33). In our present study, based on mice models, we found that GDF-15 alleviated lupus by reducing renal damage (**Figure 4**), reversing CD8^+^, CD19^+^, and T_H_2 cells dysregulation (**Figure 5**), inhibiting production of IL-1β, IL-2, IL-4, IL-21, IL-22 (**Figure 6**), ANA, and total IgG (**Figure 7**). The anti-inflammatory role of GDF-15 was also discussed in the GDF-15 gene knockout mouse model (37). Therefore, it is possible that pro-inflammatory components in SLE patients may promote GDF-15 expression, and GDF-15 may mediate a negative feedback mechanism to suppress excessive pro-inflammatory response in SLE patients like the cytokine IL-37 in inflammatory rheumatic diseases. However, the hypothesis needs to be clarified in the future. For instance, which signaling or what mechanism is involved in GDF-15 negatively regulates lupus development. Moreover, in the present study, serum levels of GDF-15 levels were positively related to SLEDAI. Serum levels of GDF-15 positively related to SLEDAI are only a statistical result in the present study. Whether GDF-15 is really related to SLEDAI needs to be confirmed in the future with larger sample sizes in multiple centers. Furthermore, functional study will help to evaluate whether GDF-15 may affect SLEDAI in the future.

There are some limitations in this study. First, serum levels of GDF-15 were analyzed in 54 SLE patients and GDF-15 gene polymorphisms were genotyped in another independent 289 SLE patients, and association of polymorphisms with serum levels of GDF-15 cannot be evaluated. Therefore, we cannot conclude whether GDF-15 gene polymorphisms affect GDF-15 expression, which may participate in lupus progression. Second, because limited number of SLE patients had cardiovascular diseases, or metabolic syndrome, or antiphospholipid syndrome, therefore, we cannot discuss whether there is difference of serum levels of GDF-15 between SLE patients with cardiovascular diseases and patients without cardiovascular diseases, patients with metabolic syndrome and patients without metabolic syndrome, and patients with antiphospholipid syndrome and patients without antiphospholipid syndrome, respectively. Because we have found a significant association of serum GDF-15 levels with some clinical and laboratory characteristics in SLE patients, functional studies are needed to discuss how GDF-15 affects the features.

In conclusion, our study showed that high level of GDF-15 was related to SLE pathogenesis, and serum GDF-15 may be a biomarker for SLE diagnosis. GDF-15 gene polymorphisms were associated with SLE risk in Chinese Han population. GDF-15 treatment relieved lupus development.

## Data Availability Statement

The raw data supporting the conclusions of this article will be made available by the authors, without undue reservation.

## Ethics Statement

The studies involving human participants were reviewed and approved by Ethic Research Committee of Affiliated Hospital of Southwest Medical University. The patients/participants provided their written informed consent to participate in this study. The animal study was reviewed and approved by Animal Ethics Committee of Southwest Medical University. Written informed consent was obtained from the individual(s) for the publication of any potentially identifiable images or data included in this article.

## Author Contributions

Study conception and design: W-DX, QH, and A-FH. Acquisition of data, analysis and interpretation of data: CY and RL. Drafting the article: W-DX, QH, and A-FH. Final approval of the version of the article to be published: all authors, and that all authors agree to be accountable for all aspects of the work.

## Funding

This work was supported by grants from the Sichuan Provincial Natural Science Foundation. (2022NSFSC0697, 2022NSFSC0694).

## Conflict of Interest

The authors declare that the research was conducted in the absence of any commercial or financial relationships that could be construed as a potential conflict of interest.

## Publisher’s Note

All claims expressed in this article are solely those of the authors and do not necessarily represent those of their affiliated organizations, or those of the publisher, the editors and the reviewers. Any product that may be evaluated in this article, or claim that may be made by its manufacturer, is not guaranteed or endorsed by the publisher.

## Supplementary Material

Supplementary Table 1 Information of primer for GDF-15 gene polymorphisms.

Supplementary Table 2 Pathological grading criteria for renal injury.

Supplementary Table 3 The Hardy-Weinberg’s expectation test in patients and controls of GDF-15 gene polymorphisms.

Supplementary Table 4 Association of GDF-15 gene polymorphisms in SLE patients with clinical, laboratory features (qualitative variables).

## Abbreviations

SLE, systemic lupus erythematosus; STAT4, signal transducer and activator of transcription 4; DAP1, death-associated protein 1; MAB2, mannose binding lectin 2; PGE2, Prostaglandins E2; PINX1, PIN2/TERF1-interacting telomerase inhibitor 1; LN, lupus nephritis; WT, wild-type; GDF-15, growth differentiation factor 15; TGF-β;, transforming growth factor-β ESR, erythrocyte sedimentation rate; ROC, receiver operating characteristic; RA, rheumatoid arthritis; OA, osteoarthritis; IFNγ, interferon-gamma.
