# Supplementary material for: GDF-15: A Potential Biomarker and Therapeutic Target in Systemic Lupus Erythematosus
Source: Front Immunol. 2022 Jul 14;13:926373. doi: 10.3389/fimmu.2022.926373 (PMC9332889; doi:10.3389/fimmu.2022.926373)
Supplement: Supplementary Table 1 — Information of primer for GDF-15 gene polymorphisms. [file Table_1.docx]

Supplementary table 1 Information of primer for GDF-15 gene polymorphisms.

| Polymorphism | Primer Allele X | Primer Allele Y | Primer Common | Allele X | Allele Y |
| --- | --- | --- | --- | --- | --- |
| rs1055150 | GCAGGACAACTGAGGTCGCC | GCAGGACAACTGAGGTCGCG | TGCATATGAGCAGTCCTGGTCCTT | C | G |
| rs1058587 | GGGCCCGAGCGGACAGTC | GGGCCCGAGCGGACAGTG | GCAGAGCGCGTGCGCGCAA | C | G |
| rs1059369 | GCCCTGTCCGGCAGATAG | CTGCCCTGTCCGGCAGATAC | CTTACCCACGCATGCCTGTCACAT | C | G |
| rs1059519 | GTGAGAGGTTTGCCGGAGTCAT | GAGAGGTTTGCCGGAGTCAC | CTCTTAGCCCGCCCTAAACCCAT | A | G |
| rs1227731 | AGGTGTCACTAACATGGAAGTGCT | GGTGTCACTAACATGGAAGTGCC | TTAGTCCCATCACCCCTCCTGTTT | A | G |
| rs4808793 | GGAGCATCTGAGAGCCATTCAC | GGAGCATCTGAGAGCCATTCAG | ACAGCCATGCCCGGGCAAGAA | C | G |
| rs16982345 | CGGAATCTGGAGTCTTCGGA | CTCGGAATCTGGAGTCTTCGGT | GTTTCCCGGGACCCTCAGAGTT | A | T |
