# Supplementary material for: GDF-15: A Potential Biomarker and Therapeutic Target in Systemic Lupus Erythematosus
Source: Front Immunol. 2022 Jul 14;13:926373. doi: 10.3389/fimmu.2022.926373 (PMC9332889; doi:10.3389/fimmu.2022.926373)
Supplement: Supplementary Table 2 — Pathological grading criteria for renal injury. [file Table_2.docx]

Supplementary table 2 Pathological grading criteria for renal injury.

| Lesion type | Degree | Score |
| --- | --- | --- |
| Glomerular injury | None, < 5% | 0 |
|  | Mild, 5%-25% | 1 |
|  | Moderate, 26%-50% | 2 |
|  | Severe, >50% | 3 |
| Tubular injury | None | 0 |
|  | Mild, swelling of renal tubular epithelium, loss of nuclei, disappearance of brush border | 1 |
|  | Moderate, focal tubular epithelial necrosis | 2 |
|  | Severe, massive necrosis | 3 |
| Renal interstitial inflammation | None, < 5% | 0 |
|  | Mild, 5%-25% | 1 |
|  | Moderate, 26%-50% | 2 |
|  | Severe, >50% | 3 |
| Renal interstitial fibrosis | None, < 5% | 0 |
|  | Mild, 5%-25% | 1 |
|  | Moderate, 26%-50% | 2 |
|  | Severe, >50% | 3 |
| Protein tubule | None, <5% | 0 |
|  | Mild, homogeneous red-stained proteins were found in renal tubules, 5%-25% | 1 |
|  | Moderate, renal tubules appear as protein tubules, 26%-50% | 2 |
|  | Severe, tubular protein type >50% | 3 |
