# Supplementary material for: GDF-15: A Potential Biomarker and Therapeutic Target in Systemic Lupus Erythematosus
Source: Front Immunol. 2022 Jul 14;13:926373. doi: 10.3389/fimmu.2022.926373 (PMC9332889; doi:10.3389/fimmu.2022.926373)
Supplement: Supplementary Table 3 — The Hardy-Weinberg’s expectation test in patients and controls of GDF-15 gene polymorphisms. [file Table_3.docx]

Supplementary table 3 The Hardy-Weinberg's expectation test in patients and controls of GDF-15 gene polymorphisms.

| Polymorphism | Position | ObsHET | PredHET | %Geno | FamTrio | MendErr | MAF | Alleles | χ^2^ | P value |
| --- | --- | --- | --- | --- | --- | --- | --- | --- | --- | --- |
| rs1055150 | 18499784 | 0.442 | 0.435 | 100.0 | 0 | 0 | 0.319 | C:G | 0.724 | 0.696 |
| rs1058587 | 18499422 | 0.430 | 0.404 | 100.0 | 0 | 0 | 0.281 | G:C | 4.902 | 0.086 |
| rs1059369 | 18497141 | 0.471 | 0.483 | 100.0 | 0 | 0 | 0.408 | A:T | 1.445 | 0.486 |
| rs1059519 | 18497024 | 0.434 | 0.431 | 100.0 | 0 | 0 | 0.314 | G:C | 0.152 | 0.927 |
| rs1227731 | 18497903 | 0.290 | 0.283 | 100.0 | 0 | 0 | 0.171 | G:A | 1.019 | 0.601 |
| rs4808793 | 18493837 | 0.441 | 0.432 | 100.0 | 0 | 0 | 0.315 | C:G | 1.012 | 0.603 |
| rs16982345 | 18500722 | 0.408 | 0.412 | 100.0 | 0 | 0 | 0.290 | G:A | 0.356 | 0.837 |

ObsHET refers to observed heterozygosityis; PredHET is predicted heterozygosity; %Geno is percentage of non-missing genotypes for the polymorphism; FamTrio refers to number of fully genotyped family trios for the polymorphism (0 for datasets with unrelated individuals); MendErr is number of observed Mendelian inheritance errors (0 for datasets with unrelated individuals); MAF means minor allele frequency (using founders only).
