# Supplementary material for: GDF-15: A Potential Biomarker and Therapeutic Target in Systemic Lupus Erythematosus
Source: Front Immunol. 2022 Jul 14;13:926373. doi: 10.3389/fimmu.2022.926373 (PMC9332889; doi:10.3389/fimmu.2022.926373)
Supplement: Supplementary Table 4 — Association of GDF-15 gene polymorphisms in SLE patients with clinical, laboratory features (qualitative variables). [file Table_4.docx]

Supplementary table 4 Association of GDF-15 gene polymorphisms in SLE patients with clinical, laboratory features (qualitative variables).

| Polymorphism | Features |  | Genotype frequency n (%) | | | P_1_ |  | Allele frequency n (%) | | P_2_ |
| --- | --- | --- | --- | --- | --- | --- | --- | --- | --- | --- |
|  |  |  | CC | CG | GG |  |  | C | G |  |
| rs1055150 | Arthritis | + | 60 (45.5) | 62 (47.0) | 10 (7.6) | 0.323 |  | 182 (68.9) | 82 (31.1) | 0.770 |
|  |  | - | 69 (43.9) | 82 (52.2) | 6 (3.8) |  |  | 220 (70.1) | 94 (29.9) |  |
|  | Discoid | + | 47 (39.5) | 68 (57.1) | 4 (3.4) | 0.078 |  | 162 (68.1) | 76 (31.9) | 0.517 |
|  |  | - | 82 (48.2) | 76 (44.7) | 12 (7.1) |  |  | 240 (70.6) | 100 (29.4) |  |
|  | Alopecia | + | 42 (45.2) | 45 (48.4) | 6 (6.5) | 0.872 |  | 129 (69.4) | 57 (30.6) | 0.944 |
|  |  | - | 87 (44.4) | 99 (50.5) | 10 (5.1) |  |  | 273 (69.6) | 119 (30.4) |  |
|  | Oral ulcers | + | 18 (42.9) | 22 (52.4) | 2 (4.8) | 0.927 |  | 58 (69.0) | 26 (31.0) | 0.914 |
|  |  | - | 111 (44.9) | 122 (49.4) | 14 (5.7) |  |  | 344 (69.6) | 150 (30.4) |  |
|  | Pleurisy | + | 7 (29.2) | 14 (58.3) | 3 (12.5) | 0.130 |  | 28 (58.3) | 20 (41.7) | 0.078 |
|  |  | - | 122 (46.0) | 130 (49.1) | 13 (4.9) |  |  | 374 (70.6) | 156 (29.4) |  |
|  | Pericarditis | + | 8 (32.0) | 15 (60.0) | 2 (8.0) | 0.397 |  | 31 (62.0) | 19 (38.0) | 0.225 |
|  |  | - | 121 (45.8) | 129 (48.9) | 14 (5.3) |  |  | 371 (70.3) | 157 (29.7) |  |
|  | Vasculitis | + | 13 (50.0) | 11 (42.3) | 2 (7.7) | 0.688 |  | 37 (71.2) | 15 (28.8) | 0.792 |
|  |  | - | 116 (44.1) | 133 (50.6) | 14 (5.3) |  |  | 365 (69.4) | 161 (30.6) |  |
|  | Fever | + | 20 (35.1) | 34 (59.6) | 3 (5.3) | 0.242 |  | 74 (64.9) | 40 (35.1) | 0.230 |
|  |  | - | 109 (47.0) | 110 (47.4) | 13 (5.6) |  |  | 328 (70.7) | 136 (29.3) |  |
|  | Hypocomplementemia | + | 64 (43.8) | 75 (51.4) | 7 (4.8) | 0.788 |  | 203 (69.5) | 89 (30.5) | 0.998 |
|  |  | - | 65 (45.5) | 69 (48.3) | 9 (6.3) |  |  | 199 (69.6) | 87 (30.4) |  |
|  | anti-dsDNA | + | 32 (47.1) | 33 (48.5) | 3 (4.4) | 0.839 |  | 97 (71.3) | 39 (28.7) | 0.607 |
|  |  | - | 97 (43.9) | 111 (50.2) | 13 (5.9) |  |  | 305 (69.0) | 137 (31.0) |  |
|  | Thrombocytopenia | + | 21 (47.7) | 21 (47.7) | 2 (4.5) | 0.883 |  | 63 (71.6) | 25 (28.4) | 0.651 |
|  |  | - | 108 (44.1) | 123 (50.2) | 14 (5.7) |  |  | 339 (69.2) | 151 (30.8) |  |
|  | Leukopenia | + | 14 (41.2) | 19 (55.9) | 1 (2.9) | 0.652 |  | 47 (69.1) | 21 (30.9) | 0.934 |
|  |  | - | 115 (45.1) | 125 (49.0) | 15 (5.9) |  |  | 355 (69.6) | 155 (30.4) |  |
|  | Hematuria | + | 45 (45.5) | 49 (49.5) | 5 (5.1) | 0.956 |  | 139 (70.2) | 59 (29.8) | 0.806 |
|  |  | - | 84 (44.2) | 95 (50.0) | 11 (5.8) |  |  | 263 (69.2) | 117 (30.8) |  |
|  | Proteinuria | + | 66 (47.1) | 65 (46.4) | 9 (6.4) | 0.496 |  | 197 (70.4) | 83 (29.6) | 0.683 |
|  |  | - | 63 (42.3) | 79 (53.0) | 7 (4.7) |  |  | 205 (68.8) | 93 (31.2) |  |
|  | ANA | + | 72 (46.8) | 73 (47.4) | 9 (5.8) | 0.640 |  | 217 (70.5) | 91 (29.5) | 0.538 |
|  |  | - | 56 (41.8) | 71 (53.0) | 7 (5.2) |  |  | 183 (68.3) | 85 (31.7) |  |
|  | anti-Sm | + | 30 (42.3) | 38 (53.5) | 3 (4.2) | 0.715 |  | 98 (69.0) | 44 (31.0) | 0.873 |
|  |  | - | 99 (45.4) | 106 (48.6) | 13 (6.0) |  |  | 304 (69.7) | 132 (30.3) |  |
|  | anti-SSA | + | 60 (48.8) | 59 (48.0) | 4 (3.3) | 0.224 |  | 179 (72.8) | 67 (27.2) | 0.148 |
|  |  | - | 69 (41.6) | 85 (51.2) | 12 (7.2) |  |  | 223 (67.2) | 109 (32.8) |  |
|  | anti-SSB | + | 22 (55.0) | 17 (42.5) | 1 (2.5) | 0.303 |  | 61 (76.3) | 19 (23.8) | 0.161 |
|  |  | - | 107 (43.0) | 127 (51.0) | 15 (6.0) |  |  | 341 (68.5) | 157 (31.5) |  |
|  | anti-RNP | + | 41 (43.6) | 48 (51.1) | 5 (5.3) | 0.957 |  | 130 (69.1) | 58 (30.9) | 0.884 |
|  |  | - | 88 (45.1) | 96 (49.2) | 11 (5.6) |  |  | 272 (69.7) | 118 (30.3) |  |
|  | SLEDAI (≥10) | + | 70 (43.8) | 83 (51.9) | 7 (4.4) | 0.538 |  | 223 (69.7) | 97 (30.3) | 0.936 |
|  |  | - | 59 (45.7) | 61 (47.3) | 9 (7.0) |  |  | 179 (69.4) | 79 (30.6) |  |
| rs1058587 |  |  | GG | GC | CC | P_1_ |  | G | C | P_2_ |
|  | Arthritis | + | 60 (45.5) | 62 (47.0) | 10 (7.6) | 0.323 |  | 182 (68.9) | 82 (31.1) | 0.605 |
|  |  | - | 69 (43.9) | 82 (52.2) | 6 (3.8) |  |  | 220 (70.1) | 94 (29.9) |  |
|  | Discoid | + | 47 (39.5) | 68 (57.1) | 4 (3.4) | 0.078 |  | 162 (68.1) | 76 (31.9) | 0.884 |
|  |  | - | 82 (48.2) | 76 (44.7) | 12 (7.1) |  |  | 240 (70.6) | 100 (29.4) |  |
|  | Alopecia | + | 42 (45.2) | 45 (48.4) | 6 (6.5) | 0.872 |  | 129 (69.4) | 57 (30.6) | 0.446 |
|  |  | - | 87 (44.4) | 99 (50.5) | 10 (5.1) |  |  | 273 (69.6) | 119 (30.4) |  |
|  | Oral ulcers | + | 18 (42.9) | 22 (52.4) | 2 (4.8) | 0.927 |  | 58 (69.0) | 26 (31.0) | 0.930 |
|  |  | - | 111 (44.9) | 122 (49.4) | 14 (5.7) |  |  | 344 (69.6) | 150 (30.4) |  |
|  | Pleurisy | + | 7 (29.2) | 14 (58.3) | 3 (12.5) | 0.130 |  | 28 (58.3) | 20 (41.7) | 0.507 |
|  |  | - | 122 (46.0) | 130 (49.1) | 13 (4.9) |  |  | 374 (70.6) | 156 (29.4) |  |
|  | Pericarditis | + | 8 (32.0) | 15 (60.0) | 2 (8.0) | 0.397 |  | 31 (62.0) | 19 (38.0) | 0.616 |
|  |  | - | 121 (45.8) | 129 (48.9) | 14 (5.3) |  |  | 371 (70.3) | 157 (29.7) |  |
|  | Vasculitis | + | 13 (50.0) | 11 (42.3) | 2 (7.7) | 0.688 |  | 37 (71.2) | 15 (28.8) | 0.752 |
|  |  | - | 116 (44.1) | 133 (50.6) | 14 (5.3) |  |  | 365 (69.4) | 161 (30.6) |  |
|  | Fever | + | 20 (35.1) | 34 (59.6) | 3 (5.3) | 0.242 |  | 74 (64.9) | 40 (35.1) | 0.447 |
|  |  | - | 109 (47.0) | 110 (47.4) | 13 (5.6) |  |  | 328 (70.7) | 136 (29.3) |  |
|  | Hypocomplementemia | + | 64 (43.8) | 75 (51.4) | 7 (4.8) | 0.788 |  | 203 (69.5) | 89 (30.5) | 0.998 |
|  |  | - | 65 (45.5) | 69 (48.3) | 9 (6.3) |  |  | 199 (69.6) | 87 (30.4) |  |
|  | anti-dsDNA | + | 32 (47.1) | 33 (48.5) | 3 (4.4) | 0.839 |  | 97 (71.3) | 39 (28.7) | 0.208 |
|  |  | - | 97 (43.9) | 111 (50.2) | 13 (5.9) |  |  | 305 (69.0) | 137 (31.0) |  |
|  | Thrombocytopenia | + | 21 (47.7) | 21 (47.7) | 2 (4.5) | 0.883 |  | 63 (71.6) | 25 (28.4) | 0.845 |
|  |  | - | 108 (44.1) | 123 (50.2) | 14 (5.7) |  |  | 339 (69.2) | 151 (30.8) |  |
|  | Leukopenia | + | 14 (41.2) | 19 (55.9) | 1 (2.9) | 0.652 |  | 47 (69.1) | 21 (30.9) | 0.918 |
|  |  | - | 115 (45.1) | 125 (49.0) | 15 (5.9) |  |  | 355 (69.6) | 155 (30.4) |  |
|  | Hematuria | + | 45 (45.5) | 49 (49.5) | 5 (5.1) | 0.956 |  | 139 (70.2) | 59 (29.8) | 0.497 |
|  |  | - | 84 (44.2) | 95 (50.0) | 11 (5.8) |  |  | 263 (69.2) | 117 (30.8) |  |
|  | Proteinuria | + | 66 (47.1) | 65 (46.4) | 9 (6.4) | 0.496 |  | 197 (70.4) | 83 (29.6) | 0.108 |
|  |  | - | 63 (42.3) | 79 (53.0) | 7 (4.7) |  |  | 205 (68.8) | 93 (31.2) |  |
|  | ANA | + | 72 (46.8) | 73 (47.4) | 9 (5.8) | 0.640 |  | 217 (70.5) | 91 (29.5) | 0.380 |
|  |  | - | 56 (41.8) | 71 (53.0) | 7 (5.2) |  |  | 183 (68.3) | 85 (31.7) |  |
|  | anti-Sm | + | 30 (42.3) | 38 (53.5) | 3 (4.2) | 0.715 |  | 98 (69.0) | 44 (31.0) | 0.873 |
|  |  | - | 99 (45.4) | 106 (48.6) | 13 (6.0) |  |  | 304 (69.7) | 132 (30.3) |  |
|  | anti-SSA | + | 60 (48.8) | 59 (48.0) | 4 (3.3) | 0.224 |  | 179 (72.8) | 67 (27.2) | 0.405 |
|  |  | - | 69 (41.6) | 85 (51.2) | 12 (7.2) |  |  | 223 (67.2) | 109 (32.8) |  |
|  | anti-SSB | + | 22 (55.0) | 17 (42.5) | 1 (2.5) | 0.303 |  | 61 (76.3) | 19 (23.8) | 0.872 |
|  |  | - | 107 (43.0) | 127 (51.0) | 15 (6.0) |  |  | 341 (68.5) | 157 (31.5) |  |
|  | anti-RNP | + | 41 (43.6) | 48 (51.1) | 5 (5.3) | 0.957 |  | 130 (69.1) | 58 (30.9) | 0.122 |
|  |  | - | 88 (45.1) | 96 (49.2) | 11 (5.6) |  |  | 272 (69.7) | 118 (30.3) |  |
|  | SLEDAI (≥10) | + | 70 (43.8) | 83 (51.9) | 7 (4.4) | 0.538 |  | 223 (69.7) | 97 (30.3) | 0.526 |
|  |  | - | 59 (45.7) | 61 (47.3) | 9 (7.0) |  |  | 179 (69.4) | 79 (30.6) |  |
| rs1059369 |  |  | AA | AT | TT | P_1_ |  | A | T | P_2_ |
|  | Arthritis | + | 42 (31.8) | 70 (53.0) | 20 (15.2) | 0.365 |  | 154 (58.3) | 110 (41.7) | 0.244 |
|  |  | - | 45 (28.7) | 78 (49.7) | 34 (21.7) |  |  | 168 (53.5) | 146 (46.5) |  |
|  | Discoid | + | 38 (31.9) | 63 (52.9) | 18 (15.1) | 0.424 |  | 139 (58.4) | 99 (41.6) | 0.275 |
|  |  | - | 49 (28.8) | 85 (50.0) | 36 (21.2) |  |  | 183 (53.8) | 157 (46.2) |  |
|  | Alopecia | + | 27 (29.0) | 54 (58.1) | 12 (12.9) | 0.152 |  | 108 (58.1) | 78 (41.9) | 0.432 |
|  |  | - | 60 (30.6) | 94 (48.0) | 42 (21.4) |  |  | 214 (54.6) | 178 (45.4) |  |
|  | Oral ulcers | + | 13 (31.0) | 22 (52.4) | 7 (16.7) | 0.936 |  | 48 (57.1) | 36 (42.9) | 0.775 |
|  |  | - | 74 (30.0) | 126 (51.0) | 47 (19.0) |  |  | 274 (55.5) | 220 (44.5) |  |
|  | Pleurisy | + | 10 (41.7) | 11 (45.8) | 3 (12.5) | 0.397 |  | 31 (64.6) | 17 (35.4) | 0.196 |
|  |  | - | 77 (29.1) | 137 (51.7) | 51 (19.2) |  |  | 291 (54.9) | 239 (45.1) |  |
|  | Pericarditis | + | 12 (48.0) | 10 (40.0) | 3 (12.0) | 0.120 |  | 34 (68.0) | 16 (32.0) | 0.067 |
|  |  | - | 75 (28.4) | 138 (52.3) | 51 (19.3) |  |  | 288 (54.5) | 240 (45.5) |  |
|  | Vasculitis | + | 7 (26.9) | 16 (61.5) | 3 (11.5) | 0.479 |  | 30 (57.7) | 22 (42.3) | 0.763 |
|  |  | - | 80 (30.4) | 132 (50.2) | 51 (19.4) |  |  | 292 (55.5) | 234 (44.5) |  |
|  | Fever | + | 24 (42.1) | 27 (47.4) | 6 (10.5) | 0.047 |  | 75 (65.8) | 39 (34.2) | 0.016 |
|  |  | - | 63 (27.2) | 121 (52.2) | 48 (20.7) |  |  | 247 (53.2) | 217 (46.8) |  |
|  | Hypocomplementemia | + | 36 (24.7) | 82 (56.2) | 28 (19.2) | 0.113 |  | 154 (52.7) | 138 (47.3) | 0.146 |
|  |  | - | 51 (35.7) | 66 (46.2) | 26 (18.2) |  |  | 168 (58.7) | 118 (41.3) |  |
|  | anti-dsDNA | + | 16 (23.5) | 40 (58.8) | 12 (17.6) | 0.311 |  | 72 (52.9) | 64 (47.1) | 0.457 |
|  |  | - | 71 (32.1) | 108 (48.9) | 42 (19.0) |  |  | 250 (56.6) | 192 (43.4) |  |
|  | Thrombocytopenia | + | 13 (29.5) | 22 (50.0) | 9 (20.5) | 0.948 |  | 48 (54.5) | 40 (45.5) | 0.811 |
|  |  | - | 74 (30.2) | 126 (51.4) | 45 (18.4) |  |  | 274 (55.9) | 216 (44.1) |  |
|  | Leukopenia | + | 8 (23.5) | 20 (58.8) | 6 (17.6) | 0.603 |  | 36 (52.9) | 32 (47.1) | 0.625 |
|  |  | - | 79 (31.0) | 128 (50.2) | 48 (18.8) |  |  | 286 (56.1) | 224 (43.9) |  |
|  | Hematuria | + | 32 (32.3) | 43 (43.4) | 24 (24.2) | 0.105 |  | 107 (54.0) | 91 (46.0) | 0.560 |
|  |  | - | 55 (28.9) | 105 (55.3) | 30 (15.8) |  |  | 215 (56.6) | 165 (43.4) |  |
|  | Proteinuria | + | 40 (28.6) | 66 (47.1) | 34 (24.3) | 0.059 |  | 146 (52.1) | 134 (47.9) | 0.094 |
|  |  | - | 47 (31.5) | 82 (55.0) | 20 (13.4) |  |  | 176 (59.1) | 122 (40.9) |  |
|  | ANA | + | 46 (29.9) | 73 (47.4) | 35 (22.7) | 0.148 |  | 165 (53.6) | 143 (46.4) | 0.339 |
|  |  | - | 40 (29.9) | 75 (56.0) | 19 (14.2) |  |  | 155 (57.8) | 113 (42.2) |  |
|  | anti-Sm | + | 15 (21.1) | 41 (57.7) | 15 (21.1) | 0.165 |  | 71 (50.0) | 71 (50.0) | 0.115 |
|  |  | - | 72 (33.0) | 107 (49.1) | 39 (17.9) |  |  | 251 (57.6) | 185 (42.4) |  |
|  | anti-SSA | + | 28 (22.8) | 66 (53.7) | 29 (23.6) | 0.033 |  | 122 (49.6) | 124 (50.4) | 0.011 |
|  |  | - | 59 (35.5) | 82 (49.4) | 25 (15.1) |  |  | 200 (60.2) | 132 (39.8) |  |
|  | anti-SSB | + | 5 (12.5) | 28 (70.0) | 7 (17.5) | 0.018 |  | 38 (47.5) | 42 (52.5) | 0.111 |
|  |  | - | 82 (32.9) | 120 (48.2) | 47 (18.9) |  |  | 284 (57.0) | 214 (43.0) |  |
|  | anti-RNP | + | 23 (24.5) | 51 (54.3) | 20 (21.3) | 0.329 |  | 97 (51.6) | 91 (48.4) | 0.167 |
|  |  | - | 64 (32.8) | 97 (49.7) | 34 (17.4) |  |  | 225 (57.7) | 165 (42.3) |  |
|  | SLEDAI (≥10) | + | 47 (29.4) | 81 (50.6) | 32 (20.0) | 0.811 |  | 175 (54.7) | 145 (45.3) | 0.582 |
|  |  | - | 40 (31.0) | 67 (51.9) | 22 (17.1) |  |  | 147 (57.0) | 111 (43.0) |  |
| rs1059519 |  |  | GG | GC | CC | P_1_ |  | G | C | P_2_ |
|  | Arthritis | + | 63 (47.7) | 59 (44.7) | 10 (7.6) | 0.187 |  | 185 (70.1) | 79 (29.9) | 0.866 |
|  |  | - | 67 (42.7) | 84 (53.5) | 6 (3.8) |  |  | 218 (69.4) | 96 (30.6) |  |
|  | Discoid | + | 46 (38.7) | 69 (58.0) | 4 (3.4) | 0.039 |  | 161 (67.6) | 77 (32.4) | 0.363 |
|  |  | - | 84 (49.4) | 74 (43.5) | 12 (7.1) |  |  | 242 (71.2) | 98 (28.8) |  |
|  | Alopecia | + | 41 (44.1) | 47 (50.5) | 5 (5.4) | 0.970 |  | 129 (69.4) | 57 (30.6) | 0.894 |
|  |  | - | 89 (45.4) | 96 (49.0) | 11 (5.6) |  |  | 274 (69.9) | 118 (30.1) |  |
|  | Oral ulcers | + | 19 (45.2) | 21 (50.0) | 2 (4.8) | 0.972 |  | 59 (70.2) | 25 (29.8) | 0.912 |
|  |  | - | 111 (44.9) | 122 (49.4) | 14 (5.7) |  |  | 344 (69.6) | 150 (30.4) |  |
|  | Pleurisy | + | 7 (29.2) | 13 (54.2) | 4 (16.7) | 0.024 |  | 27 (56.3) | 21 (43.8) | 0.034 |
|  |  | - | 123 (46.4) | 130 (49.1) | 12 (4.5) |  |  | 376 (70.9) | 154 (29.1) |  |
|  | Pericarditis | + | 8 (32.0) | 15 (60.0) | 2 (8.0) | 0.380 |  | 31 (62.0) | 19 (38.0) | 0.214 |
|  |  | - | 122 (46.2) | 128 (48.5) | 14 (5.3) |  |  | 372 (70.5) | 156 (29.5) |  |
|  | Vasculitis | + | 13 (50.0) | 11 (42.3) | 2 (7.7) | 0.706 |  | 37 (71.2) | 15 (28.8) | 0.814 |
|  |  | - | 117 (44.5) | 132 (50.2) | 14 (5.3) |  |  | 366 (69.6) | 160 (30.4) |  |
|  | Fever | + | 20 (35.1) | 35 (61.4) | 2 (3.5) | 0.128 |  | 75 (65.8) | 39 (34.2) | 0.308 |
|  |  | - | 110 (47.4) | 108 (46.6) | 14 (6.0) |  |  | 328 (70.7) | 136 (29.3) |  |
|  | Hypocomplementemia | + | 63 (43.2) | 76 (52.1) | 7 (4.8) | 0.635 |  | 202 (69.2) | 90 (30.8) | 0.773 |
|  |  | - | 67 (46.9) | 67 (46.9) | 9 (6.3) |  |  | 201 (70.3) | 85 (29.7) |  |
|  | anti-dsDNA | + | 30 (44.1) | 35 (51.5) | 3 (4.4) | 0.865 |  | 95 (69.9) | 41 (30.1) | 0.970 |
|  |  | - | 100 (45.2) | 108 (48.9) | 13 (5.9) |  |  | 308 (69.7) | 134 (30.3) |  |
|  | Thrombocytopenia | + | 20 (45.5) | 23 (52.3) | 1 (2.3) | 0.582 |  | 63 (71.6) | 25 (28.4) | 0.679 |
|  |  | - | 110 (44.9) | 120 (49.0) | 15 (6.1) |  |  | 340 (69.4) | 150 (30.6) |  |
|  | Leukopenia | + | 13 (38.2) | 19 (55.9) | 2 (5.9) | 0.699 |  | 45 (66.2) | 23 (33.8) | 0.498 |
|  |  | - | 117 (45.9) | 124 (48.6) | 14 (5.5) |  |  | 358 (70.2) | 152 (29.8) |  |
|  | Hematuria | + | 43 (43.4) | 51 (51.5) | 5 (5.1) | 0.874 |  | 137 (69.2) | 61 (30.8) | 0.841 |
|  |  | - | 87 (45.8) | 92 (48.4) | 11 (5.8) |  |  | 266 (70.0) | 114 (30.0) |  |
|  | Proteinuria | + | 64 (45.7) | 67 (47.9) | 9 (6.4) | 0.753 |  | 195 (69.6) | 85 (30.4) | 0.968 |
|  |  | - | 66 (44.3) | 76 (51.0) | 7 (4.7) |  |  | 208 (69.8) | 90 (30.2) |  |
|  | ANA | + | 72 (46.8) | 73 (47.4) | 9 (5.8) | 0.715 |  | 217 (70.5) | 91 (29.5) | 0.604 |
|  |  | - | 57 (42.5) | 70 (52.2) | 7 (5.2) |  |  | 184 (68.7) | 84 (31.3) |  |
|  | anti-Sm | + | 30 (42.3) | 39 (54.9) | 2 (2.8) | 0.372 |  | 99 (69.7) | 43 (30.3) | 0.999 |
|  |  | - | 100 (45.9) | 104 (47.7) | 14 (6.4) |  |  | 304 (69.7) | 132 (30.3) |  |
|  | anti-SSA | + | 60 (48.8) | 59 (48.0) | 4 (3.3) | 0.246 |  | 179 (72.8) | 67 (27.2) | 0.171 |
|  |  | - | 70 (42.2) | 84 (50.6) | 12 (7.2) |  |  | 224 (67.5) | 108 (32.5) |  |
|  | anti-SSB | + | 24 (60.0) | 15 (37.5) | 1 (2.5) | 0.108 |  | 63 (78.8) | 17 (21.3) | 0.058 |
|  |  | - | 106 (42.6) | 128 (51.4) | 15 (6.0) |  |  | 340 (68.3) | 158 (31.7) |  |
|  | anti-RNP | + | 39 (41.5) | 51 (54.3) | 4 (4.3) | 0.489 |  | 129 (68.6) | 59 (31.4) | 0.688 |
|  |  | - | 91 (46.7) | 92 (47.2) | 12 (6.2) |  |  | 274 (70.3) | 116 (29.7) |  |
|  | SLEDAI (≥10) | + | 68 (42.5) | 85 (53.1) | 7 (4.4) | 0.312 |  | 221 (69.1) | 99 (30.9) | 0.700 |
|  |  | - | 62 (48.1) | 58 (45.0) | 9 (7.0) |  |  | 182 (70.5) | 76 (29.5) |  |
| rs1227731 |  |  | GG | GA | AA | P_1_ |  | G | A | P_2_ |
|  | Arthritis | + | 86 (65.2) | 42 (31.8) | 4 (3.0) | 0.240 |  | 214 (81.1) | 50 (18.9) | 0.203 |
|  |  | - | 111 (70.7) | 45 (28.7) | 1 (0.6) |  |  | 267 (85.0) | 47 (15.0) |  |
|  | Discoid | + | 74 (62.2) | 43 (36.1) | 2 (1.7) | 0.164 |  | 191 (80.3) | 47 (19.7) | 0.110 |
|  |  | - | 123 (72.4) | 44 (25.9) | 3 (1.8) |  |  | 290 (85.3) | 50 (14.7) |  |
|  | Alopecia | + | 63 (67.7) | 29 (31.2) | 1 (1.1) | 0.964 |  | 155 (83.3) | 31 (16.7) | 0.959 |
|  |  | - | 134 (68.4) | 58 (29.6) | 4 (2.0) |  |  | 326 (83.2) | 66 (16.8) |  |
|  | Oral ulcers | + | 25 (59.5) | 16 (38.1) | 1 (2.4) | 0.305 |  | 66 (78.6) | 18 (21.4) | 0.218 |
|  |  | - | 172 (69.6) | 71 (28.7) | 4 (1.6) |  |  | 415 (84.0) | 79 (16.0) |  |
|  | Pleurisy | + | 14 (58.3) | 9 (37.5) | 1 (4.2) | 0.226 |  | 37 (77.1) | 11 (22.9) | 0.235 |
|  |  | - | 183 (69.1) | 78 (29.4) | 4 (1.5) |  |  | 444 (83.8) | 86 (16.2) |  |
|  | Pericarditis | + | 15 (60.0) | 10 (40.0) | 0 (0.0) | 0.590 |  | 40 (80.0) | 10 (20.0) | 0.524 |
|  |  | - | 182 (68.9) | 77 (29.2) | 5 (1.9) |  |  | 441 (83.5) | 87 (16.5) |  |
|  | Vasculitis | + | 19 (73.1) | 7 (26.9) | 0 (0.0) | 0.888 |  | 45 (86.5) | 7 (13.5) | 0.502 |
|  |  | - | 178 (67.7) | 80 (30.4) | 5 (1.9) |  |  | 436 (82.9) | 90 (17.1) |  |
|  | Fever | + | 32 (56.1) | 25 (43.9) | 0 (0.0) | 0.037 |  | 89 (78.1) | 25 (21.9) | 0.101 |
|  |  | - | 165 (71.1) | 62 (26.7) | 5 (2.2) |  |  | 392 (84.5) | 72 (15.5) |  |
|  | Hypocomplementemia | + | 101 (69.2) | 44 (30.1) | 1 (0.7) | 0.495 |  | 246 (84.2) | 46 (15.8) | 0.504 |
|  |  | - | 96 (67.1) | 43 (30.1) | 4 (2.8) |  |  | 235 (82.2) | 51 (17.8) |  |
|  | anti-dsDNA | + | 46 (67.6) | 22 (32.4) | 0 (0.0) | 0.615 |  | 114 (83.8) | 22 (16.2) | 0.829 |
|  |  | - | 151 (68.3) | 65 (29.4) | 5 (2.3) |  |  | 367 (83.0) | 75 (17.0) |  |
|  | Thrombocytopenia | + | 28 (63.6) | 16 (36.4) | 0 (0.0) | 0.472 |  | 72 (81.8) | 16 (18.2) | 0.703 |
|  |  | - | 169 (69.0) | 71 (29.0) | 5 (2.0) |  |  | 409 (83.5) | 81 (16.5) |  |
|  | Leukopenia | + | 22 (64.7) | 11 (32.4) | 1 (2.9) | 0.532 |  | 55 (80.9) | 13 (19.1) | 0.583 |
|  |  | - | 175 (68.6) | 76 (29.8) | 4 (1.6) |  |  | 426 (83.5) | 84 (16.5) |  |
|  | Hematuria | + | 68 (68.7) | 30 (30.3) | 1 (1.0) | 0.926 |  | 166 (83.8) | 32 (16.2) | 0.773 |
|  |  | - | 129 (67.9) | 57 (30.0) | 4 (2.1) |  |  | 315 (82.9) | 65 (17.1) |  |
|  | Proteinuria | + | 93 (66.4) | 46 (32.9) | 1 (0.7) | 0.307 |  | 232 (82.9) | 48 (17.1) | 0.822 |
|  |  | - | 104 (69.8) | 41 (27.5) | 4 (2.7) |  |  | 249 (83.6) | 49 (16.4) |  |
|  | ANA | + | 110 (71.4) | 42 (27.3) | 2 (1.3) | 0.406 |  | 262 (85.1) | 46 (14.9) | 0.179 |
|  |  | - | 86 (64.2) | 45 (33.6) | 3 (2.2) |  |  | 217 (81.0) | 51 (19.0) |  |
|  | anti-Sm | + | 50 (70.4) | 21 (29.6) | 0 (0.0) | 0.597 |  | 121 (85.2) | 21 (14.8) | 0.464 |
|  |  | - | 147 (67.4) | 66 (30.3) | 5 (2.3) |  |  | 360 (82.6) | 76 (17.4) |  |
|  | anti-SSA | + | 92 (74.8) | 30 (24.4) | 1 (0.8) | 0.088 |  | 214 (87.0) | 32 (13.0) | 0.037 |
|  |  | - | 105 (63.3) | 57 (34.3) | 4 (2.4) |  |  | 267 (80.4) | 65 (19.6) |  |
|  | anti-SSB | + | 33 (82.5) | 7 (17.5) | 0 (0.0) | 0.117 |  | 73 (91.3) | 7 (8.8) | 0.038 |
|  |  | - | 164 (65.9) | 80 (32.1) | 5 (2.0) |  |  | 408 (81.9) | 90 (18.1) |  |
|  | anti-RNP | + | 64 (68.1) | 29 (30.9) | 1 (1.1) | 0.965 |  | 157 (83.5) | 31 (16.5) | 0.896 |
|  |  | - | 133 (68.2) | 58 (29.7) | 4 (2.1) |  |  | 324 (83.1) | 66 (16.9) |  |
|  | SLEDAI (≥10) | + | 107 (66.9) | 52 (32.5) | 1 (0.6) | 0.213 |  | 266 (83.1) | 54 (16.9) | 0.947 |
|  |  | - | 90 (69.8) | 35 (27.1) | 4 (3.1) |  |  | 215 (83.3) | 43 (16.7) |  |
| rs4808793 |  |  | CC | CG | GG | P_1_ |  | C | G | P_2_ |
|  | Arthritis | + | 61 (46.2) | 60 (45.5) | 11 (8.3) | 0.146 |  | 182 (68.9) | 82 (31.1) | 0.965 |
|  |  | - | 66 (42.0) | 85 (54.1) | 6 (3.8) |  |  | 217 (69.1) | 97 (30.9) |  |
|  | Discoid | + | 44 (37.0) | 71 (59.7) | 4 (3.4) | 0.018 |  | 159 (66.8) | 79 (33.2) | 0.333 |
|  |  | - | 83 (48.8) | 74 (43.5) | 13 (7.6) |  |  | 240 (70.6) | 100 (29.4) |  |
|  | Alopecia | + | 39 (41.9) | 48 (51.6) | 6 (6.5) | 0.819 |  | 126 (67.7) | 60 (32.3) | 0.644 |
|  |  | - | 88 (44.9) | 97 (49.5) | 11 (5.6) |  |  | 273 (69.6) | 119 (30.4) |  |
|  | Oral ulcers | + | 18 (42.9) | 22 (52.4) | 2 (4.8) | 0.920 |  | 58 (69.0) | 26 (31.0) | 0.997 |
|  |  | - | 109 (44.1) | 123 (49.8) | 15 (6.1) |  |  | 341 (69.0) | 153 (31.0) |  |
|  | Pleurisy | + | 7 (29.2) | 13 (54.2) | 4 (16.7) | 0.038 |  | 27 (56.3) | 21 (43.8) | 0.045 |
|  |  | - | 120 (45.3) | 132 (49.8) | 13 (4.9) |  |  | 372 (70.2) | 158 (29.8) |  |
|  | Pericarditis | + | 8 (32.0) | 15 (60.0) | 2 (8.0) | 0.444 |  | 31 (62.0) | 19 (38.0) | 0.261 |
|  |  | - | 119 (45.1) | 130 (49.2) | 15 (5.7) |  |  | 368 (69.7) | 160 (30.3) |  |
|  | Vasculitis | + | 13 (50.0) | 11 (42.3) | 2 (7.7) | 0.687 |  | 37 (71.2) | 15 (28.8) | 0.729 |
|  |  | - | 114 (43.3) | 134 (51.0) | 15 (5.7) |  |  | 362 (68.8) | 164 (31.2) |  |
|  | Fever | + | 19 (33.3) | 35 (61.4) | 3 (5.3) | 0.161 |  | 73 (64.0) | 41 (36.0) | 0.198 |
|  |  | - | 108 (46.6) | 110 (47.4) | 14 (6.0) |  |  | 326 (70.3) | 138 (29.7) |  |
|  | Hypocomplementemia | + | 62 (42.5) | 76 (52.1) | 8 (5.5) | 0.804 |  | 200 (68.5) | 92 (31.5) | 0.777 |
|  |  | - | 65 (45.5) | 69 (48.3) | 9 (6.3) |  |  | 199 (69.6) | 87 (30.4) |  |
|  | anti-dsDNA | + | 29 (42.6) | 35 (51.5) | 4 (5.9) | 0.969 |  | 93 (68.4) | 43 (31.6) | 0.852 |
|  |  | - | 98 (44.3) | 110 (49.8) | 13 (5.9) |  |  | 306 (69.2) | 136 (30.8) |  |
|  | Thrombocytopenia | + | 20 (45.5) | 22 (50.0) | 2 (4.5) | 0.912 |  | 62 (70.5) | 26 (29.5) | 0.754 |
|  |  | - | 107 (43.7) | 123 (50.2) | 15 (6.1) |  |  | 337 (68.8) | 153 (31.2) |  |
|  | Leukopenia | + | 13 (38.2) | 19 (55.9) | 2 (5.9) | 0.765 |  | 45 (66.2) | 23 (33.8) | 0.588 |
|  |  | - | 114 (44.7) | 126 (49.4) | 15 (5.9) |  |  | 354 (69.4) | 156 (30.6) |  |
|  | Hematuria | + | 45 (45.5) | 48 (48.5) | 6 (6.1) | 0.918 |  | 138 (69.7) | 60 (30.3) | 0.803 |
|  |  | - | 82 (43.2) | 97 (51.1) | 11 (5.8) |  |  | 261 (68.7) | 119 (31.3) |  |
|  | Proteinuria | + | 65 (46.4) | 65 (46.4) | 10 (7.1) | 0.392 |  | 195 (69.6) | 85 (30.4) | 0.758 |
|  |  | - | 62 (41.6) | 80 (53.7) | 7 (4.7) |  |  | 204 (68.5) | 94 (31.5) |  |
|  | ANA | + | 72 (46.8) | 72 (46.8) | 10 (6.5) | 0.422 |  | 216 (70.1) | 92 (29.9) | 0.470 |
|  |  | - | 54 (40.3) | 73 (54.5) | 7 (5.2) |  |  | 181 (67.5) | 87 (32.5) |  |
|  | anti-Sm | + | 31 (43.7) | 37 (52.1) | 3 (4.2) | 0.774 |  | 99 (69.7) | 43 (30.3) | 0.838 |
|  |  | - | 96 (44.0) | 108 (49.5) | 14 (6.4) |  |  | 300 (68.8) | 136 (31.2) |  |
|  | anti-SSA | + | 60 (48.8) | 59 (48.0) | 4 (3.3) | 0.145 |  | 179 (72.8) | 67 (27.2) | 0.095 |
|  |  | - | 67 (40.4) | 86 (51.8) | 13 (7.8) |  |  | 220 (66.3) | 112 (33.7) |  |
|  | anti-SSB | + | 24 (60.0) | 15 (37.5) | 1 (2.5) | 0.078 |  | 63 (78.8) | 17 (21.3) | 0.043 |
|  |  | - | 103 (41.4) | 130 (52.2) | 16 (6.4) |  |  | 336 (67.5) | 162 (32.5) |  |
|  | anti-RNP | + | 40 (42.6) | 49 (52.1) | 5 (5.3) | 0.886 |  | 129 (68.6) | 59 (31.4) | 0.811 |
|  |  | - | 87 (44.6) | 96 (49.2) | 12 (6.2) |  |  | 270 (69.2) | 120 (30.8) |  |
|  | SLEDAI (≥10) | + | 69 (43.1) | 83 (51.9) | 8 (5.0) | 0.692 |  | 221 (69.1) | 99 (30.9) | 0.986 |
|  |  | - | 58 (45.0) | 62 (48.1) | 9 (7.0) |  |  | 178 (69.0) | 80 (31.0) |  |
| rs16982345 |  |  | GG | GA | AA | P_1_ |  | G | A | P_2_ |
|  | Arthritis | + | 61 (46.2) | 55 (41.7) | 16 (12.1) | 0.554 |  | 177 (67.0) | 87 (33.0) | 0.265 |
|  |  | - | 82 (52.2) | 60 (38.2) | 15 (9.6) |  |  | 224 (71.3) | 90 (28.7) |  |
|  | Discoid | + | 64 (53.8) | 45 (37.8) | 10 (8.4) | 0.373 |  | 173 (72.7) | 65 (27.3) | 0.148 |
|  |  | - | 79 (46.5) | 70 (41.2) | 21 (12.4) |  |  | 228 (67.1) | 112 (32.9) |  |
|  | Alopecia | + | 42 (45.2) | 41 (44.1) | 10 (10.8) | 0.562 |  | 125 (67.2) | 61 (32.8) | 0.435 |
|  |  | - | 101 (51.5) | 74 (37.8) | 21 (10.7) |  |  | 276 (70.4) | 116 (29.6) |  |
|  | Oral ulcers | + | 21 (50.0) | 18 (42.9) | 3 (7.1) | 0.702 |  | 60 (71.4) | 24 (28.6) | 0.659 |
|  |  | - | 122 (49.4) | 97 (39.3) | 28 (11.3) |  |  | 341 (69.0) | 153 (31.0) |  |
|  | Pleurisy | + | 16 (66.7) | 6 (25.0) | 2 (8.3) | 0.208 |  | 38 (79.2) | 10 (20.8) | 0.124 |
|  |  | - | 127 (47.9) | 109 (41.1) | 29 (10.9) |  |  | 363 (68.5) | 167 (31.5) |  |
|  | Pericarditis | + | 11 (44.0) | 10 (40.0) | 4 (16.0) | 0.645 |  | 32 (64.0) | 18 (36.0) | 0.388 |
|  |  | - | 132 (50.0) | 105 (39.8) | 27 (10.2) |  |  | 369 (69.9) | 159 (30.1) |  |
|  | Vasculitis | + | 11 (42.3) | 12 (46.2) | 3 (11.5) | 0.739 |  | 34 (65.4) | 18 (34.6) | 0.513 |
|  |  | - | 132 (50.2) | 103 (39.2) | 28 (10.6) |  |  | 367 (69.8) | 159 (30.2) |  |
|  | Fever | + | 22 (38.6) | 29 (50.9) | 6 (10.5) | 0.143 |  | 73 (64.0) | 41 (36.0) | 0.167 |
|  |  | - | 121 (52.2) | 86 (37.1) | 25 (10.8) |  |  | 328 (70.7) | 136 (29.3) |  |
|  | Hypocomplementemia | + | 79 (54.1) | 54 (37.0) | 13 (8.9) | 0.250 |  | 212 (72.6) | 80 (27.4) | 0.089 |
|  |  | - | 64 (44.8) | 61 (42.7) | 18 (12.6) |  |  | 189 (66.1) | 97 (33.9) |  |
|  | anti-dsDNA | + | 41 (60.3) | 23 (33.8) | 4 (5.9) | 0.089 |  | 105 (77.2) | 31 (22.8) | 0.024 |
|  |  | - | 102 (46.2) | 92 (41.6) | 27 (12.2) |  |  | 296 (67.0) | 146 (33.0) |  |
|  | Thrombocytopenia | + | 19 (43.2) | 17 (38.6) | 8 (18.2) | 0.210 |  | 55 (62.5) | 33 (37.5) | 0.128 |
|  |  | - | 124 (50.6) | 98 (40.0) | 23 (9.4) |  |  | 346 (70.6) | 144 (29.4) |  |
|  | Leukopenia | + | 16 (47.1) | 17 (50.0) | 1 (2.9) | 0.199 |  | 49 (72.1) | 19 (27.9) | 0.610 |
|  |  | - | 127 (49.8) | 98 (38.4) | 30 (11.8) |  |  | 352 (69.0) | 158 (31.0) |  |
|  | Hematuria | + | 54 (54.5) | 31 (31.3) | 14 (14.1) | 0.077 |  | 139 (70.2) | 59 (29.8) | 0.756 |
|  |  | - | 89 (46.8) | 84 (44.2) | 17 (8.9) |  |  | 262 (68.9) | 118 (31.1) |  |
|  | Proteinuria | + | 71 (50.7) | 55 (39.3) | 14 (10.0) | 0.889 |  | 197 (70.4) | 83 (29.6) | 0.620 |
|  |  | - | 72 (48.3) | 60 (40.3) | 17 (11.4) |  |  | 204 (68.5) | 94 (31.5) |  |
|  | ANA | + | 80 (51.9) | 61 (39.6) | 13 (8.4) | 0.450 |  | 221 (71.8) | 87 (28.2) | 0.283 |
|  |  | - | 63 (47.0) | 54 (40.3) | 17 (12.7) |  |  | 180 (67.2) | 88 (32.8) |  |
|  | anti-Sm | + | 39 (54.9) | 26 (36.6) | 6 (8.5) | 0.533 |  | 104 (73.2) | 38 (26.8) | 0.250 |
|  |  | - | 104 (47.7) | 89 (40.8) | 25 (11.5) |  |  | 297 (68.1) | 139 (31.9) |  |
|  | anti-SSA | + | 65 (52.8) | 48 (39.0) | 10 (8.1) | 0.393 |  | 178 (72.4) | 68 (27.6) | 0.181 |
|  |  | - | 78 (47.0) | 67 (40.4) | 21 (12.7) |  |  | 223 (67.2) | 109 (32.8) |  |
|  | anti-SSB | + | 19 (47.5) | 17 (42.5) | 4 (10.0) | 0.930 |  | 55 (68.8) | 25 (31.3) | 0.896 |
|  |  | - | 124 (49.8) | 98 (39.4) | 27 (10.8) |  |  | 346 (69.5) | 152 (30.5) |  |
|  | anti-RNP | + | 56 (59.6) | 30 (31.9) | 8 (8.5) | 0.058 |  | 142 (75.5) | 46 (24.5) | 0.026 |
|  |  | - | 87 (44.6) | 85 (43.6) | 23 (11.8) |  |  | 259 (66.4) | 131 (33.6) |  |
|  | SLEDAI (≥10) | + | 82 (51.3) | 60 (37.5) | 18 (11.3) | 0.673 |  | 224 (70.0) | 96 (30.0) | 0.717 |
|  |  | - | 61 (47.3) | 55 (42.6) | 13 (10.1) |  |  | 177 (68.6) | 81 (31.4) |  |

P_1_: 3×2 contingency table.

P_2_: 2×2 contingency table.
